# Global Effects of the Developmental Regulator BldB in Streptomyces venezuelae

**DOI:** 10.1128/jb.00135-23

**Published:** 2023-05-30

**Authors:** Marieta M. Avramova, Clare E. M. Stevenson, Govind Chandra, Neil A. Holmes, Matthew J. Bush, Kim C. Findlay, Mark J. Buttner

**Affiliations:** a Department of Molecular Microbiology, John Innes Centre, Norwich, United Kingdom; b Department of Biochemistry and Metabolism, John Innes Centre, Norwich, United Kingdom; c Department of Cell and Developmental Biology, John Innes Centre, Norwich, United Kingdom; Geisel School of Medicine at Dartmouth

**Keywords:** *Streptomyces*, morphological differentiation, sporulation, transcriptional regulation

## Abstract

In *Streptomyces*, the Bld (Bald) regulators control formation of the reproductive aerial hyphae. The functions of some of these regulators have been well characterized, but BldB has remained enigmatic. In addition to the *bldB* gene itself, Streptomyces venezuelae has 10 paralogs of *bldB* that sit next to paralogs of *whiJ* and *abaA*. Transcriptome sequencing (RNA-seq) revealed that loss of BldB function causes the dramatic transcriptional upregulation of the *abaA* paralogs and a novel inhibitor of sporulation, *iosA*, and that cooverexpression of just two of these genes, *iosA* and *abaA6*, was sufficient to recapitulate the *bldB* mutant phenotype. Further RNA-seq analysis showed that the transcription factor WhiJ9 is required for the activation of *iosA* seen in the *bldB* mutant, and biochemical studies showed that WhiJ9 mediates the activation of *iosA* expression by binding to direct repeats in the *iosA-whiJ9* intergenic region. BldB and BldB9 hetero-oligomerize, providing a potential link between BldB and the *iosA-whiJ9-bldB9* locus. This work greatly expands our overall understanding of the global effects of the BldB developmental regulator.

**IMPORTANCE** To reproduce and disperse, the filamentous bacterium *Streptomyces* develops specialized reproductive structures called aerial hyphae. The formation of these structures is controlled by the *bld* (bald) genes, many of which encode transcription factors whose functions have been characterized. An exception is BldB, a protein whose biochemical function is unknown. In this study, we gain insight into the global effects of BldB function by examining the genome-wide transcriptional effects of deleting *bldB*. We identify a small set of genes that are dramatically upregulated in the absence of BldB. We show that their overexpression causes the *bldB* phenotype and characterize a transcription factor that mediates the upregulation of one of these target genes. Our results provide new insight into how BldB influences *Streptomyces* development.

## INTRODUCTION

*Streptomyces* species are primarily soil-dwelling, filamentous bacteria that have a complex developmental life cycle during which they transition from vegetative growth to a reproductive phase associated with the production of specialized aerial hyphae that differentiate into dispersive exospores through a synchronized septation event (1–4). In addition to their fascinating developmental life cycle, *Streptomyces* bacteria are also notable as the most abundant source of antibiotics and other natural products used in medicine (5–7). Differentiation and antibiotic production are temporally and genetically coordinated, and so understanding the signals and mechanisms that initiate these processes is of significant interest. The key regulators that control the developmental pathway fall into two classes (1–3). Bld (bald) regulators are required for the formation of the hair-like reproductive structures, and so mutations in *bld* loci result in a “bald” phenotype. Whi (white) regulators are required for the differentiation of aerial hyphae into mature spores, and mutations in *whi* loci therefore prevent synthesis of the polyketide spore pigment that normally gives mature colonies their characteristic color. The first four *bld* genes to be identified were *bldA*, *bldB*, *bldC*, and *bldD* (8), and BldA, BldC, and BldD have been well characterized functionally (9–17). In contrast, BldB has remained something of a mystery. In Streptomyces coelicolor, where *bldB* was first discovered, null mutations confer profound defects not only on the formation of aerial hyphae but also on the production of antibiotics (8, 18–20). BldB is a small acidic protein (pI 4.33) of 10.8 kDa that has been shown to homodimerize (18–21), but its biochemical function has remained obscure.

**FIG 1:**
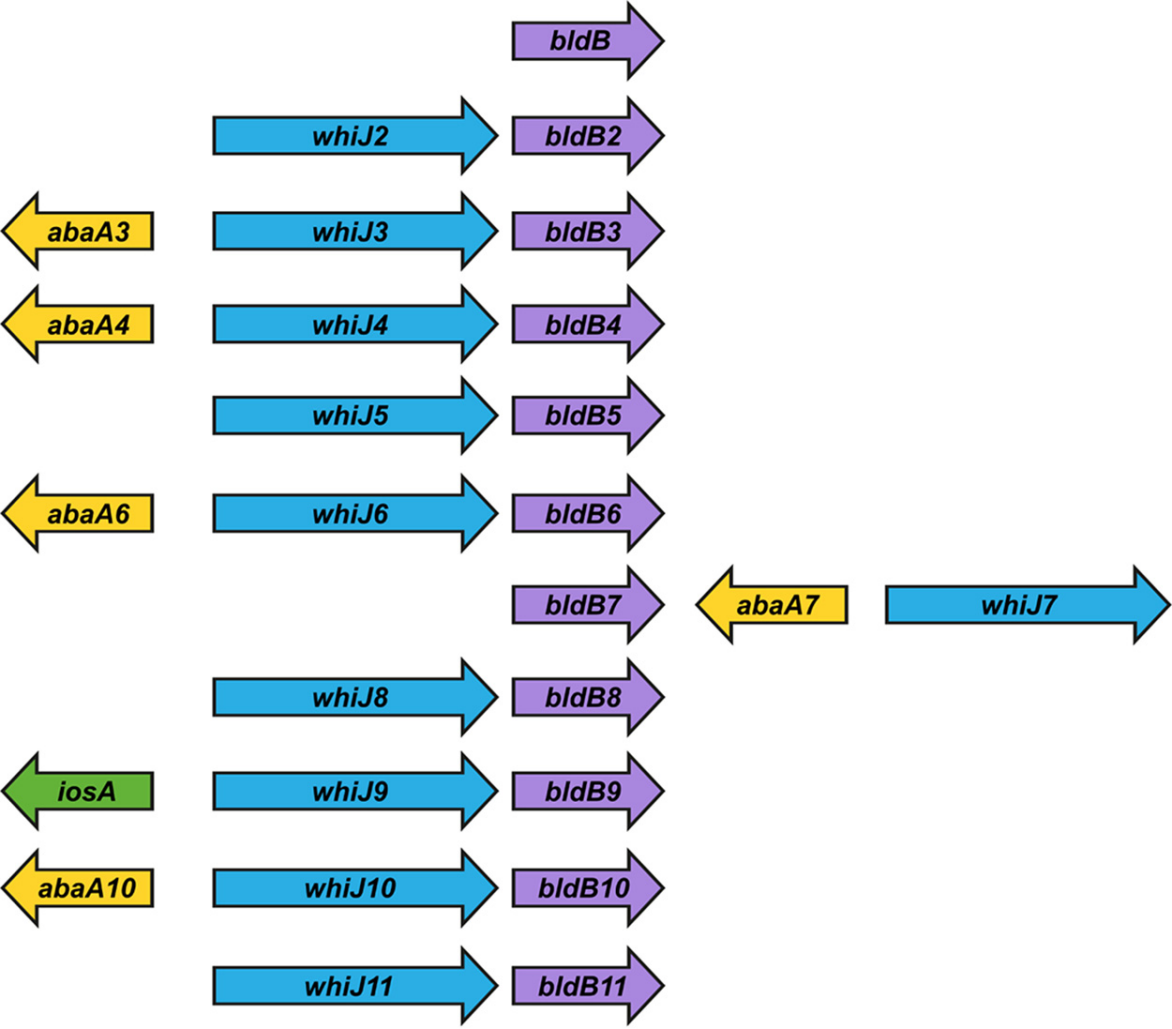
Genomic arrangement of the *bldB*, *whiJ*, and *abaA* paralogs in *S. venezuelae*. An arrow chart representing the genomic arrangement of the canonical *bldB* gene, the *bldB* paralogs (*bldB2* to *bldB11*), and the adjacent *whiJ* and *abaA* paralogs, plus *iosA*, is shown. Gene lengths are not to scale. The *vnz* identifiers for the *bldB* paralogs are as follows: *bldB*, *vnz26620*; *bldB2*, *vnz15145*; *bldB3*, *vnz29075*; *bldB4*, *vnz25555*; *bldB5*, *vnz20565*; *bldB6*, *vnz09895*; *bldB7*, *vnz16140*; *bldB8*, *vnz28285*; *bldB9*, *vnz16680*; *bldB10*, *vnz31505*; *bldB11*, *vnz28375*.

A further complication is that most individual *Streptomyces* species encode numerous paralogs of BldB. For example, in S. coelicolor, there are 23 paralogous genes in addition to the canonical *bldB* gene itself (*sco5723*) (20). Notably, across different *Streptomyces* species, these noncanonical BldB paralogs are usually found encoded next to paralogs of AbaA (OrfA) (having an HATPase_c domain, a domain associated with certain anti-sigma factors) and paralogs of WhiJ (a predicted DNA-binding protein with an N-terminal helix-turn-helix [HTH]-Xre domain) (Fig. 1). Several studies have described aspects of this syntenic relationship (19, 20, 22–27). *abaA* was first described in a study in which the overexpression of a 5-gene locus resulted in increased production of the antibiotic actinorhodin in S. coelicolor. For this reason, the locus was called *abaA* (antibiotic biosynthesis activator A), with the five genes designated *orfA* to *orfE* (28). *whiJ* was originally defined by several poorly characterized sporulation-defective white-colony mutants of S. coelicolor (29). Complementation of those mutants identified the gene to be *sco4543*, named *whiJ*, encoding a putative DNA-binding protein (25). The *whiJ* locus was also independently identified by transposon mutagenesis (22). Unexpectedly, although some mutations in *whiJ* gave a white-colony sporulation defect, complete deletion of *whiJ* in S. coelicolor gave a wild-type (WT) morphology (25). Like *bldB*, *abaA* (*orfA*), and *whiJ* are members of large paralogous gene sets in most *Streptomyces* species (22–26).

Here, we analyze BldB in Streptomyces venezuelae, now well established as a model species for the study of development in the genus *Streptomyces* (30). In addition to the canonical *bldB* gene (*vnz26620*), *S. venezuelae* has 10 *bldB* paralogs, which we designated *bldB2* to *bldB11* (Fig. 1). As in other species, the genetic context of these 10 paralogs in *S. venezuelae* is striking. Each is located close to a *whiJ* paralog, and five of them also have a paralog of *abaA* (*orfA*) nearby (referred to simply as *abaA* from here on). The only *bldB* paralog that does not lie close to paralogs of *whiJ* or *abaA* is the canonical *bldB* gene itself (Fig. 1). We show that loss of BldB function causes the dramatic transcriptional upregulation of all five *abaA* paralogs and of a novel inhibitor of sporulation, *iosA*, and that cooverexpression of just two of these genes, *iosA* and *abaA6*, is sufficient to recapitulate the *bldB* mutant phenotype. We go on to show that the transcription factor WhiJ9 is required for the activation of *iosA* seen in the *bldB* mutant and that WhiJ9 mediates the activation of *iosA* expression by binding to direct repeats in the *iosA-whiJ9* intergenic region. We also show that BldB and BldB9 hetero-oligomerize, providing a potential link between BldB and the *iosA-whiJ9-bldB9* locus.

## RESULTS

### Construction and characterization of an *S. venezuelae bldB* mutant.

**FIG 2:**
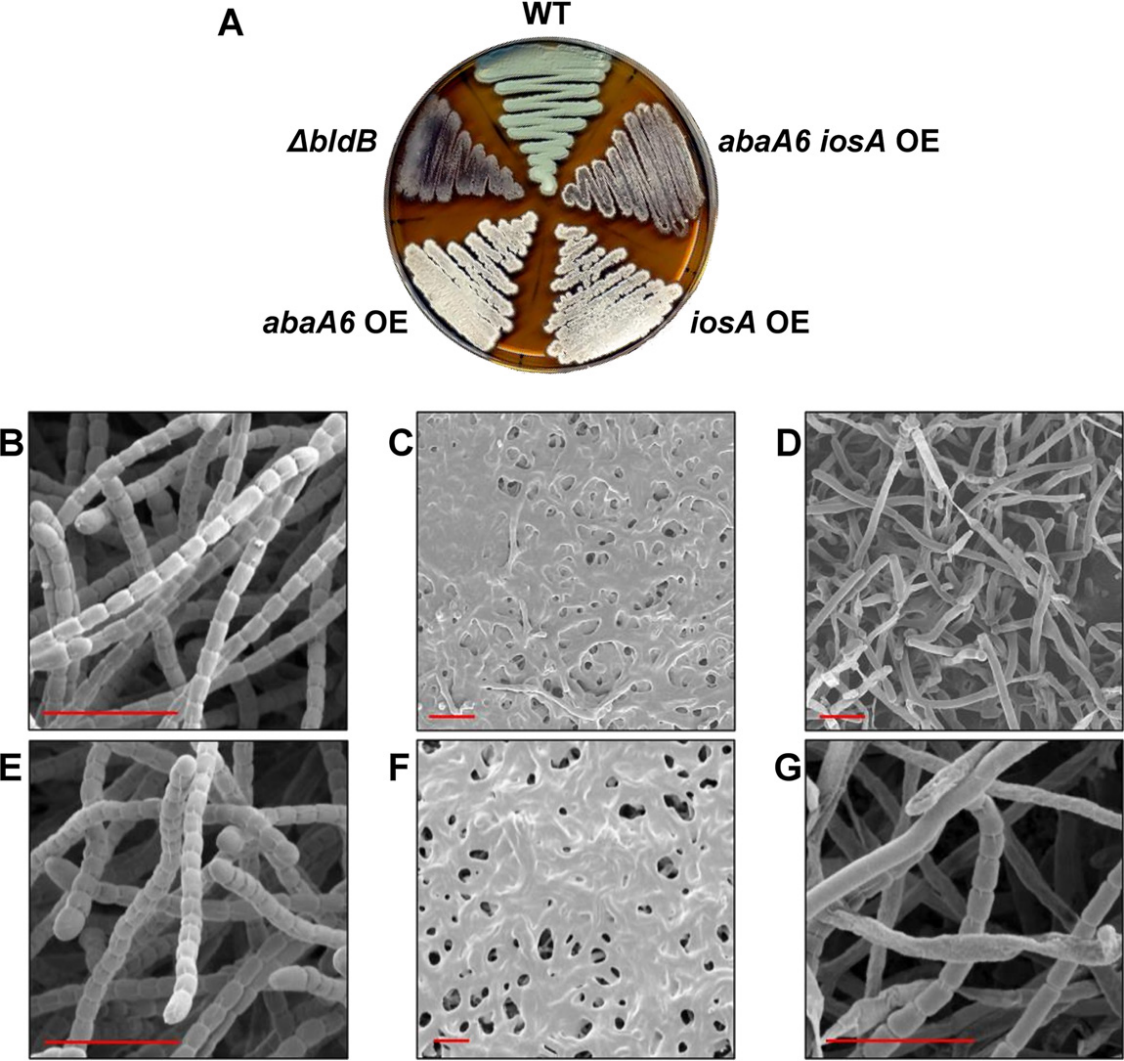
Phenotypic characterization of the Δ*bldB* mutant and the *abaA6 iosA* double-overexpression (OE) strain. (A) Plate showing the phenotypes of WT *S. venezuelae*, the *bldB* mutant, and the single- and double-overexpression strains for *abaA6* and *iosA*, grown for 3 days at 28°C on MYM agar. (B to G) SEMs comparing the phenotypes of WT *S. venezuelae* (B), the colony center of the Δ*bldB* mutant (C), the colony periphery of the Δ*bldB* mutant (D), the WT carrying the pIJ10257 overexpression plasmid (empty vector control) (E), the colony center of the *abaA6 iosA* double-overexpression strain (F), and the colony periphery of the *abaA6 iosA* double-overexpression strain (G). Scale bars in red, 5 μm.

An *S. venezuelae* Δ*bldB*::*apr* mutant was constructed, and its phenotype was analyzed. While the 2-day-old Δ*bldB* strain was completely bald on maltose yeast extract malt extract (MYM) agar, the phenotype was somewhat leaky, with the formation of sparse aerial hyphae after 3 days of incubation (Fig. 2A). Scanning electron microscopy (SEM) of single colonies of the *bldB* mutant showed that the center of each colony was bald (Fig. 2C). while the periphery of the Δ*bldB* colony exhibited some aerial hyphae with sporadic sporulation (Fig. 2D). However, these mostly undifferentiated aerial hyphae looked unhealthy; some were lysed, and their appearance was not comparable to that of the aerial hyphae of WT *S. venezuelae* (Fig. 2B). Normal sporulation was restored to the *S. venezuelae bldB* mutant by introducing a single copy of the WT *bldB* gene under the control of its native promoter, expressed in *trans* from the ΦBT1 integration site (see Fig. S1 in the supplemental material). Thus, BldB appears to play similar roles in *S. venezuelae* and S. coelicolor.

### Loss of BldB function causes dramatic transcriptional upregulation of the *abaA* paralogs and *iosA*.

**FIG 3:**
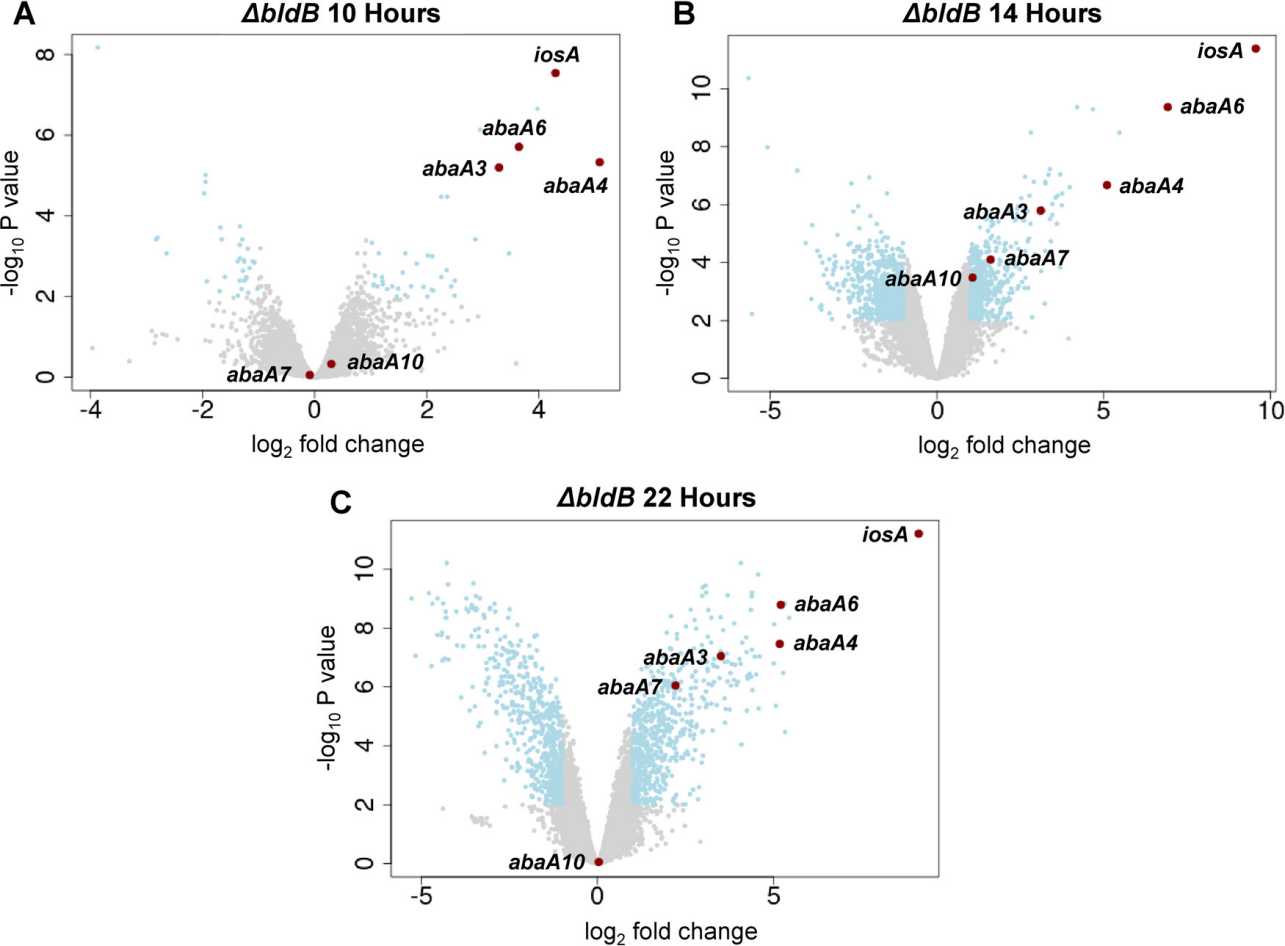
Differential gene expression in the Δ*bldB* mutant compared to WT *S. venezuelae* at 10 h (A), 14 h (B), and 22 h (C) of growth in liquid MYM. Volcano plots were generated to illustrate the differential expression in the Δ*bldB* mutant. The *x* axes represent the log_2_ fold change of gene expression in the Δ*bldB* mutant, compared to WT *S. venezuelae.* The *y* axes represent the −log_10_ *P* value. Represented by blue dots are all significantly down- or upregulated genes with a log_2_ fold change of less than −1 or above 1, respectively, and a *P* value of ≤0.01. The *abaA* paralogs and *iosA* are highlighted in red.

We used transcriptome sequencing (RNA-seq) to determine which genes are differentially expressed in the Δ*bldB* mutant compared to WT *S. venezuelae* and might therefore contribute to the *bldB* phenotype. The strains were grown in MYM liquid medium, and RNA was isolated at 10, 14, and 22 h postinoculation. The resulting data are presented as volcano plots in Fig. 3. The most striking finding was the dramatic upregulation of the five *abaA* paralogs in the Δ*bldB* mutant at one or more of the time points. One additional gene, *vnz16670*, here named *iosA* (see below), was the most highly upregulated gene across the whole data set at the 14- and 22-h time points, with a log_2_ fold change of 9.56 at 14 h, meaning that expression of *iosA* was ~750 times higher in the Δ*bldB* mutant than in the WT (Fig. 3B). Notably, the *iosA* gene lies adjacent to *whiJ9* and *bldB9*, in the same genomic context as the *abaA* paralogs (Fig. 1). However, despite its genomic context, IosA is not a paralog of AbaA. IosA is described further in Discussion.

### Overexpression of *abaA6* or *iosA* results in a sporulation-deficient phenotype.

Because the five *abaA* paralogs and *iosA* were among the most highly upregulated genes in the Δ*bldB* mutant, and given their genomic context next to *bldB* paralogs, it seemed likely that their overexpression might contribute significantly to the *bldB* phenotype. To investigate this possibility, we generated overexpression strains for all six genes by placing them individually under the control of the strong constitutive ermE* promoter by using the integrative plasmid pIJ10257. Individual overexpression of four of the genes had no obvious developmental consequences (Fig. S2), but strikingly, individual overexpression of either *abaA6* or *iosA* in WT *S. venezuelae* resulted in a white phenotype (Fig. 2A; Fig. S2). For this reason, we named *vnz16670 iosA* (for inhibitor of sporulation A).

### Combined overexpression of *abaA6* and *iosA* recapitulates the Δ*bldB* phenotype.

*abaA6* and *iosA* were the two most dramatically upregulated genes in the Δ*bldB* mutant (Fig. 3). Given that the individual overexpression of either *abaA6* or *iosA* led to a white phenotype, we wondered if cooverexpressing *abaA6* and *iosA* in WT *S. venezuelae* would result in a phenotype that more closely resembled that of the Δ*bldB* mutant. We therefore constructed a strain in which both genes were overexpressed under the same ermE* promoter, with a ribosome binding site inserted between the two genes, as described by Gallagher et al. (31). The resulting double-overexpression strain appeared bald and resembled the Δ*bldB* mutant much more closely than the single-overexpression strains, which were white (Fig. 2A). Under the electron microscope, the Δ*bldB* mutant and the *abaA6 iosA* double-overexpression strains looked very similar, with only vegetative mycelium present in the center of the colonies (compare Fig. 2C and F) and sporadic sporulation in the periphery of the colonies (compare Fig. 2D and G). Thus, the double overexpression of just two genes, *abaA6* and *iosA*, was sufficient to recapitulate the Δ*bldB* phenotype.

### BldB is not a DNA-binding protein.

The biochemical function of BldB is unknown. BldB is not similar to any characterized DNA-binding protein and does not contain a recognized DNA-binding motif. However, the dramatic overexpression of the *abaA* paralogs and *iosA* in the *bldB* mutant was potentially consistent with the idea that BldB is a repressor of these genes. In addition, Streptomyces lividans BldB has been suggested to bind its own promoter, based on an *in vitro* electrophoretic mobility shift assay (EMSA) (21), although EMSAs are well known to produce artifacts. We tried to demonstrate an interaction between *S. venezuelae* BldB and its own promoter by using the surface plasmon resonance (SPR)-based ReDCaT method (32, 33). The sequence of the *S. venezuelae bldB* promoter was split into a set of 40-bp overlapping oligonucleotides, and the oligonucleotides were bound to a streptavidin SPR chip via a biotinylated DNA linker to test for BldB binding. BldB did not bind any of the assayed oligonucleotides.

To rigorously address the possibility that BldB might be a DNA-binding protein, we performed *in vivo* genome-wide chromatin immunoprecipitation sequencing (ChIP-seq) experiments with polyclonal anti-BldB antibody and WT *S. venezuelae*, using the Δ*bldB* mutant as the negative control. Western blot analysis across a developmental time course of the WT grown in MYM liquid sporulation medium showed that BldB was present throughout development but was most abundant in mid- to late development (Fig. S3). Based on this analysis, we chose three time points (10, 14, and 22 h postinoculation) to perform ChIP-seq, and ChIP-seq at each time point was performed in duplicate. In these experiments, we saw no evidence that BldB binds DNA *in vivo*. Taken together, these data strongly suggest that *S. venezuelae* BldB is not a DNA-binding protein.

### WhiJ9 binds to direct repeats in the *iosA-whiJ9* intergenic region.

**FIG 4:**
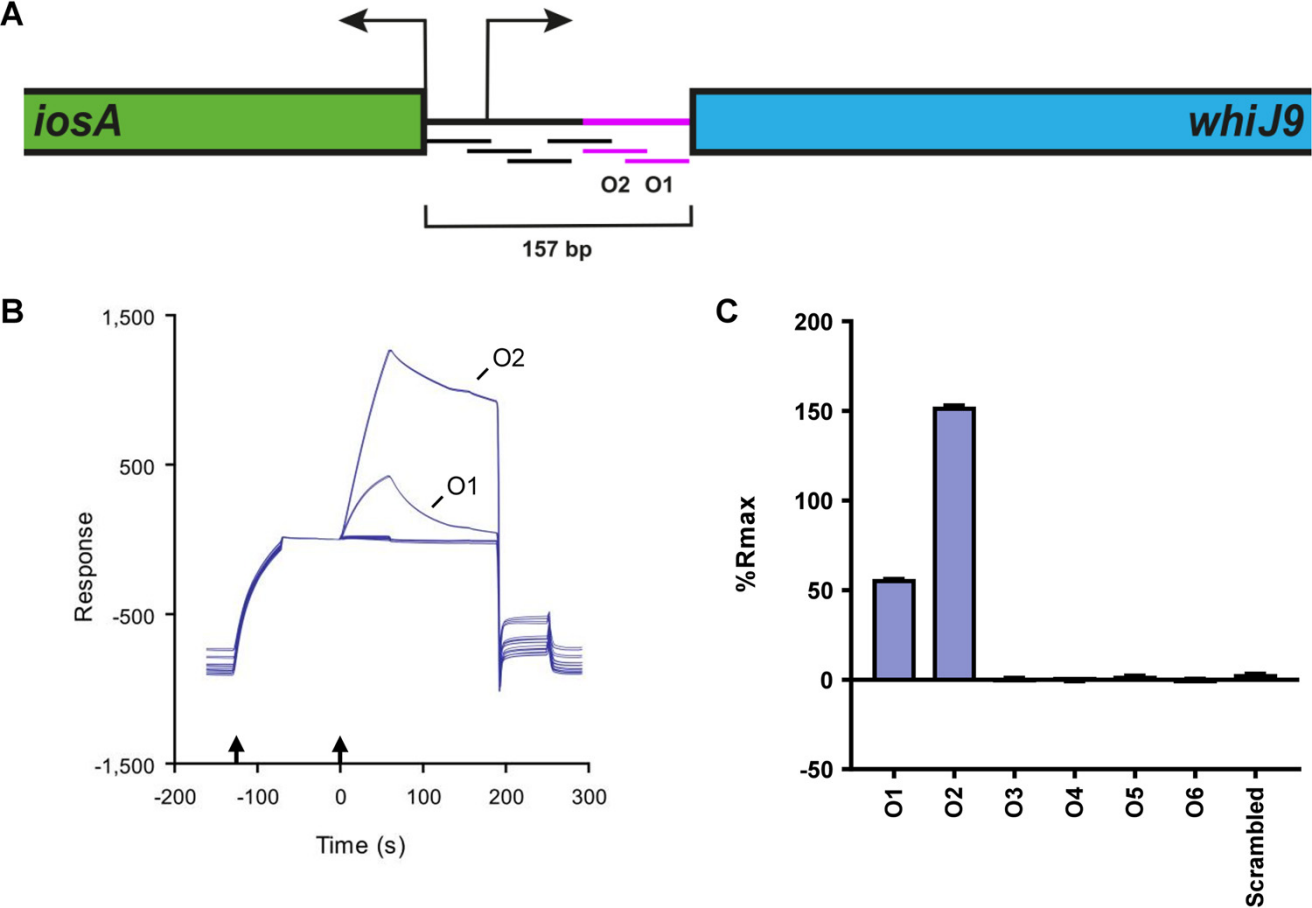
Binding of 6×His-WhiJ9 to six overlapping oligonucleotides from the *iosA-whiJ9* intergenic region. (A) Representation of the *iosA-whiJ9* intergenic region drawn to scale. The arrows represent the experimentally determined transcription start sites for *iosA* and *whiJ9*. *iosA* has a leaderless message, so the transcription start site coincides with the start codon. The six overlapping oligonucleotides used in the ReDCaT experiment are represented as lines under the intergenic region, shown in black for O3 to O6 (which did not bind WhiJ9) and pink for O1 and O2 (which did bind WhiJ9). (B) SPR sensorgram measuring binding response (*y* axis) over time (*x* axis). The oligonucleotides that did not show binding were O3 to O6 and the negative control. The two vertical arrows indicate the injections of DNA and then 6×His-WhiJ9. (C) Bar chart showing the level of 6×His-WhiJ9 binding to DNA, expressed as a percentage of the theoretical maximum response (%*R*_max_), assuming that one monomer of WhiJ9 is bound to one dsDNA molecule. Bars and error bars represent the mean and the standard error of the mean of the results of three replicates.

Because BldB is not a DNA-binding protein, we reasoned that it cannot directly control the expression of the *abaA* paralogs and *iosA*. Given the genomic context of the WhiJ paralogs, and the fact that they share an N-terminal HTH Xre DNA-binding motif, they seemed likely candidates for direct regulators of the *abaA* paralogs and *iosA*. We investigated this possibility, focusing on WhiJ9 as a potential regulator of *iosA*, because *iosA* was the most upregulated gene in the *bldB* mutant. We tested the ability of WhiJ9 to bind to the *iosA-whiJ9* intergenic region, again using the SPR-derived ReDCaT method (32, 33). The sequence of the *iosA-whiJ9* intergenic region was split into six overlapping 40-bp oligonucleotides, as indicated in Fig. 4A, and the oligonucleotides were bound to a streptavidin SPR chip via a biotinylated DNA linker to test for WhiJ9 binding. WhiJ9 specifically bound to oligonucleotides O1 and O2 in the promoter region (Fig. 4B), with a higher binding affinity for O2 (Fig. 4C).

**FIG 5:**
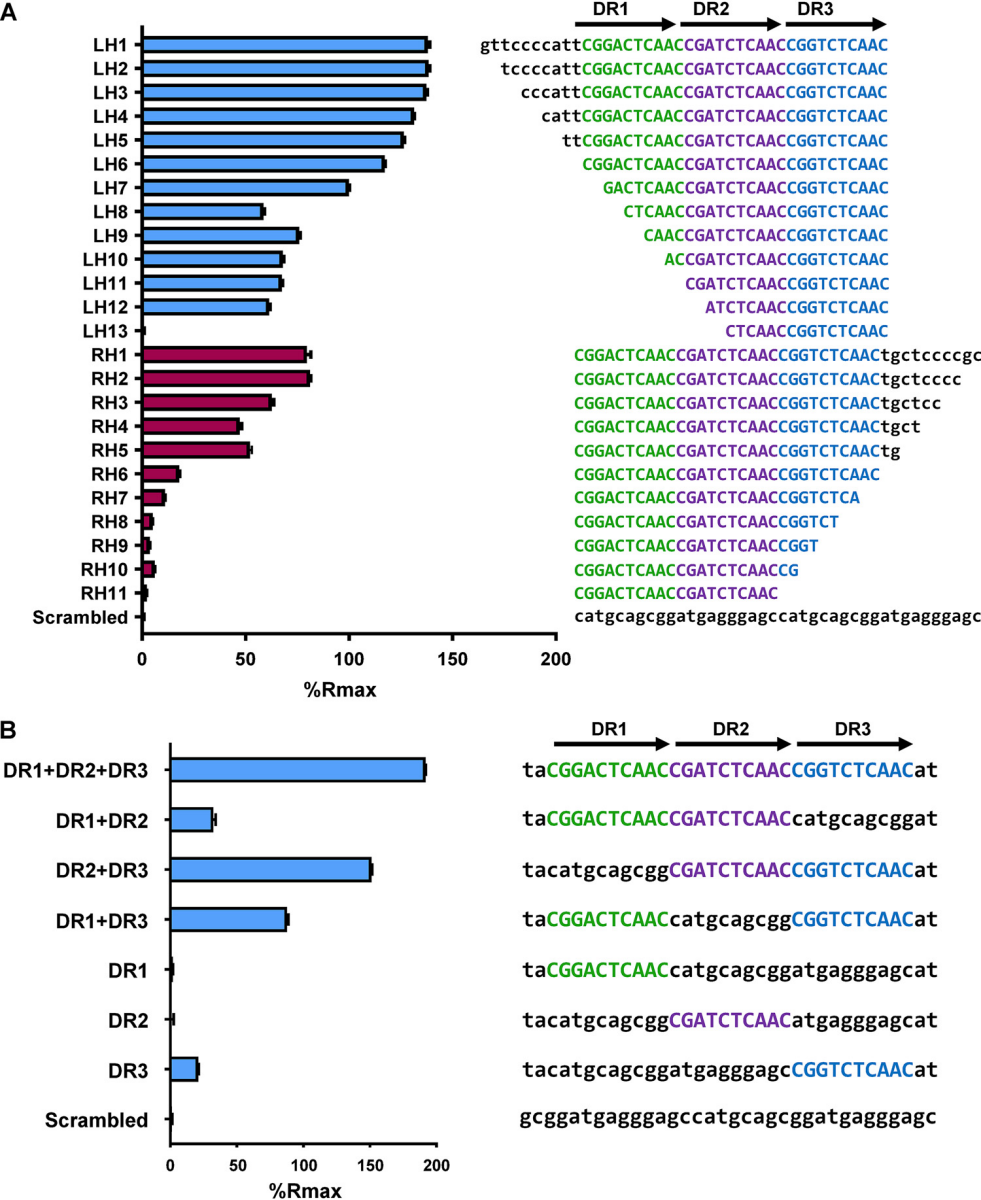
(A) 6×His-WhiJ9 “footprinting” using SPR. SPR was used to measure the binding affinity of 6×His-WhiJ9 to dsDNA oligonucleotides of various lengths from the *iosA-whiJ9* intergenic region. The bar chart on the left shows the level of 6×His-WhiJ9 binding to the DNA sequences shown on the right, expressed as a percentage of the theoretical maximum response (%*R*_max_), assuming that one monomer of WhiJ9 bound to one dsDNA molecule. Bars represent the average results of three replicates. Error bars represent the standard error of the mean. The three direct repeats are indicated with arrows and are highlighted in green (DR1), purple (DR2), and blue (DR3). (B) Binding of 6×His-WhiJ9 to the DR1, DR2, and DR3 direct repeats. SPR was used to measure the binding affinity of 6×His-WhiJ9 to 34-bp dsDNA oligonucleotides from its promoter region containing 1, 2, or all 3 direct repeats. The bar chart on the left shows the level of 6×His-WhiJ9 binding to the DNA sequences on the right, expressed as a percentage of the theoretical maximum response (%*R*_max_), assuming that one monomer of WhiJ9 bound to one dsDNA molecule. Bars represent the average results of three replicates, and error bars represent the standard error of the mean. The three direct repeats are indicated by arrows and are highlighted in green (DR1), purple (DR2), and blue (DR3).

These initial SPR experiments suggested that the WhiJ9 binding site sits at the intersection of the two overlapping oligonucleotides, O1 and O2. Inspection of this region identified 3 direct repeats, designated DR1, DR2, and DR3, with a consensus sequence of CGXXCTCAAC (Fig. 5A). The region spanning the WhiJ9 binding site was truncated 2 bp at a time from either the left or right end, and WhiJ9 binding to each sequence was assayed (Fig. 5A). WhiJ9 bound strongly to oligonucleotides LH1 and RH1, which contained all three direct repeats. When truncation was from the left end, removal of all or part of DR1 caused WhiJ9 to bind with lower affinity, resulting in a lower percentage of the theoretical maximum response (%*R*_max_) (oligonucleotides LH7 to LH11) (Fig. 5A), but did not abolish DNA binding. DNA binding was lost only when the first 4 bp of DR2 were additionally removed (oligonucleotide LH13) (Fig. 5A). In contrast, when DNA was removed from the right end, any truncation of the DR3 repeat (oligonucleotides RH7 to RH11) caused a severe drop in WhiJ9 binding, and even removal of the flanking DNA (oligonucleotides RH3 to RH6) affected binding, suggesting that the DR3 repeat was critical (Fig. 5A).

To analyze the contribution of each direct repeat, we systematically replaced one or two repeats at a time with a random DNA sequence to yield seven DNA sequences of the same length, containing combinations of one, two, or all three repeats (Fig. 5B). WhiJ9 bound the oligonucleotide containing all three direct repeats with the highest affinity but was able to bind the oligonucleotide containing DR2 and DR3 almost as well, showing that DR1 made only a minor contribution to binding. The oligonucleotide containing DR1 and DR3 (with a random DNA sequence replacing DR2) also bound WhiJ9 strongly. WhiJ9 did not bind when only DR1 or DR2 was present, but there was binding when only DR3 was present, confirming the importance of the DR3 repeat (Fig. 5B).

### WhiJ9 is required for the activation of *iosA* seen in the Δ*bldB* single mutant.

We constructed a Δ*whiJ9* Δ*bldB* double mutant and performed RNA-seq, reasoning that if WhiJ9 mediates the striking activation of *iosA* seen in the *bldB* mutant, it would be attenuated by the additional deletion of *whiJ9* in the *bldB* background. The Δ*whiJ9* Δ*bldB* double mutant had the same developmental phenotype as the Δ*bldB* single mutant.

**FIG 6:**
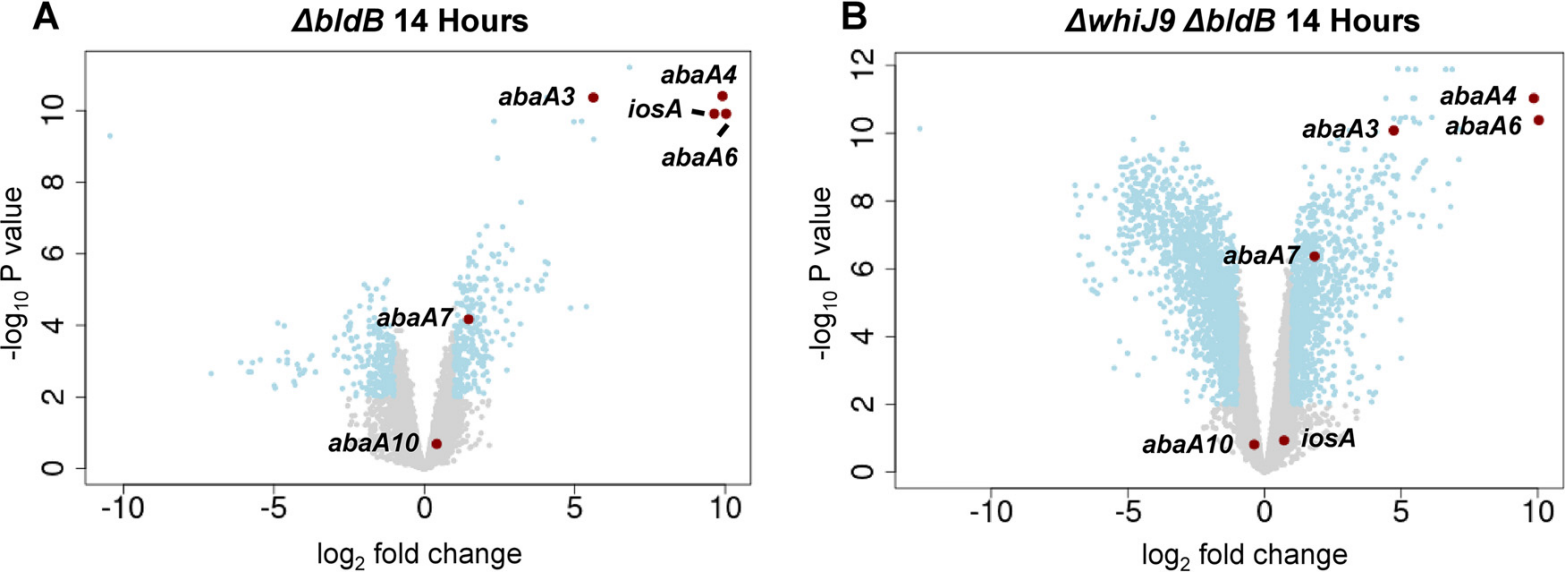
Differential gene expression in the Δ*bldB* mutant (A) and the Δ*whiJ9* Δ*bldB* double mutant (B). Volcano plots were generated to illustrate the differential expression in the Δ*bldB* mutant and the Δ*whiJ9* Δ*bldB* double mutant compared to WT *S. venezuelae* at 14 h of growth in MYM liquid medium. The *x* axes represent the log_2_ fold change of gene expression in the mutants, compared to the WT. The *y* axes represent the −log_10_ *P* value. Represented by blue dots are all significantly down- or upregulated genes with a log_2_ fold change of less than −1 or above 1, respectively, and a *P* value of ≤0.01. The *abaA* paralogs and *iosA* are highlighted in red.

RNA-seq was performed on WT *S. venezuelae*, the Δ*bldB* single mutant, and the Δ*whiJ9* Δ*bldB* double mutant in parallel. We had already performed RNA-seq analyses on WT *S. venezuelae* and the Δ*bldB* mutant (Fig. 3) but repeated them so that we could directly compare those data sets with the one from the Δ*whiJ9* Δ*bldB* double mutant. For this experiment, we used a 14-h postinoculation time point, because it was the time point at which *iosA* overexpression had been most extreme in the *bldB* single mutant (Fig. 3). We once again observed the impressive upregulation of the *abaA* paralogs and *iosA* in the Δ*bldB* mutant (Fig. 6A). In the Δ*whiJ9* Δ*bldB* double mutant (Fig. 6B), the expression of the *abaA* paralogs was as strikingly elevated as it was in the Δ*bldB* single mutant. However, the dramatic upregulation of *iosA* seen in the *bldB* single mutant was abolished in the Δ*whiJ9* Δ*bldB* double mutant (fold change of 1.65 compared to a fold change of >790 in the Δ*bldB* mutant) (Fig. 6B), demonstrating that WhiJ9 is required for the *in vivo* activation of *iosA* seen in the Δ*bldB* single mutant.

### Overexpression of WhiJ9 causes extreme overexpression of *iosA* and a white phenotype.

**FIG 7:**
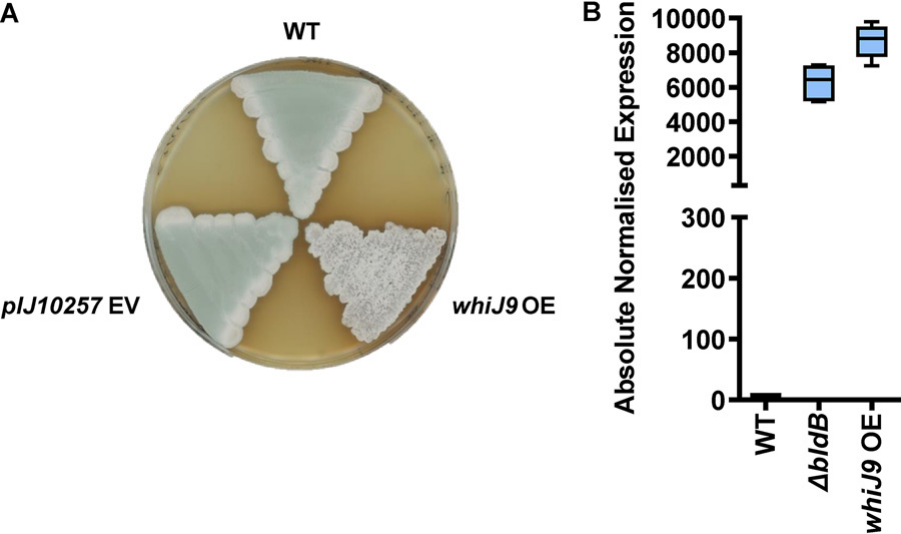
Overexpression of WhiJ9 causes extreme overexpression of *iosA* and a white phenotype. (A) Plate showing the white phenotype of the *whiJ9* overexpression strain compared to the wild type and the pIJ10257 empty vector (EV) control. The image was taken after 3 days of growth on MYM at 28°C. (B) Box-and-whiskers plot representing the absolute quantification of *iosA* expression as assayed by qRT-PCR. Differences in primer efficiency were corrected based on curves from genomic DNA amplification. Expression values for each sample were calculated relative to the *hrdB* reference and normalized to the WT value, which was set to 1. Plots represent the lower quartile (bottom of box), median (horizontal line in box), and upper quartile (top of box) of the results of two independent experiments with three technical replicates per experiment. The whiskers represent the minimum and maximum expression values.

Given that overexpression of *iosA* causes a white phenotype and that WhiJ9 mediates the activation of *iosA* in the *bldB* mutant, we looked to see if overexpression of WhiJ9 would cause a similar phenotype. *whiJ9* was placed under the control of the constitutive ermE* promoter, using the integrative plasmid pIJ10257, and the resulting construct was introduced into WT *S. venezuelae.* The *whiJ9* overexpression strain produced was indeed white on MYM agar (Fig. 7A).

Using reverse transcription-quantitative PCR (qRT-PCR), we also looked to see if the overexpression of WhiJ9 caused overexpression of *iosA*, examining the *bldB* mutant and the WT in parallel as controls. The results once again confirmed the dramatic upregulation of *iosA* in the Δ*bldB* mutant, but notably, the level of *iosA* expression was even higher in the *whiJ9* overexpression strain than in the Δ*bldB* mutant, demonstrating that overexpression of WhiJ9 causes extreme overexpression of *iosA* (Fig. 7B).

### BldB hetero-oligomerizes with its paralogs, including BldB9.

**FIG 8:**
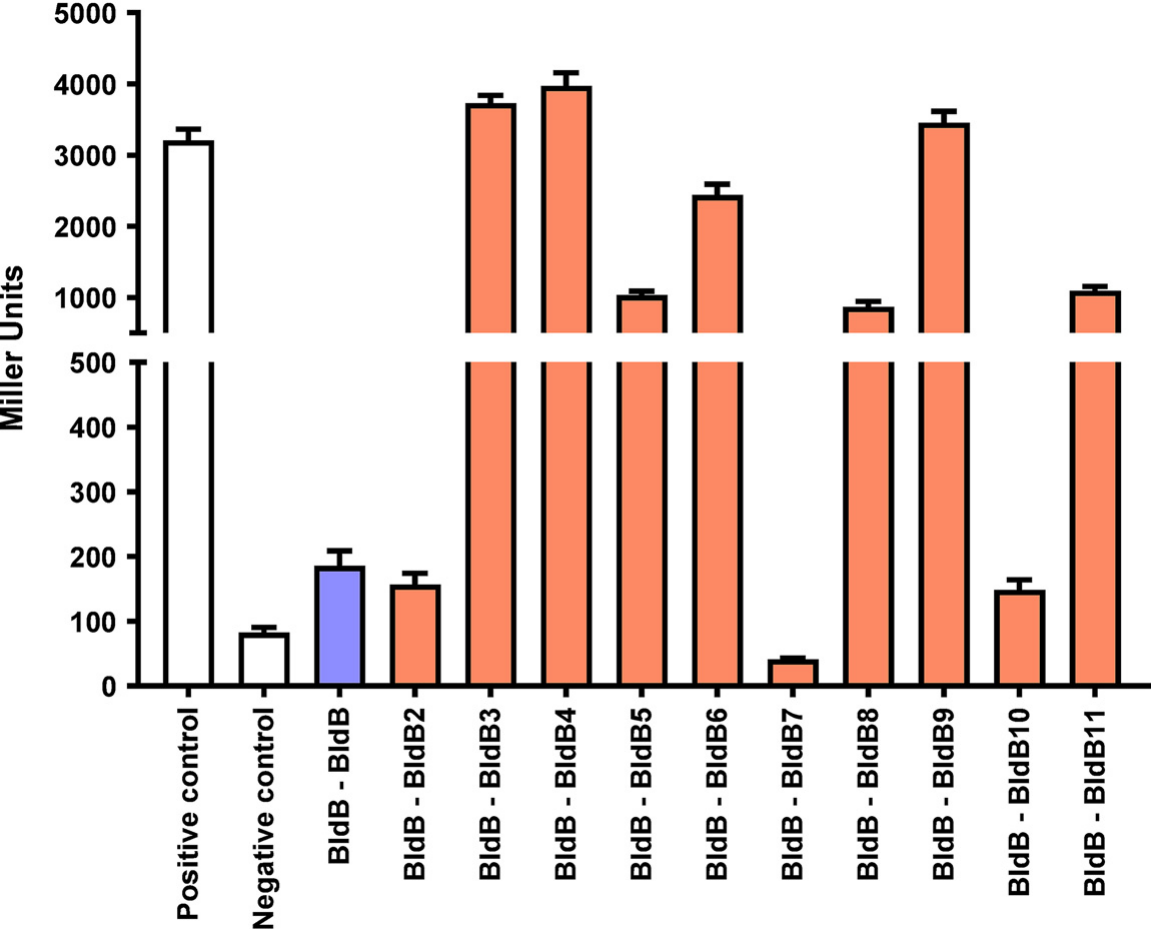
Interactions between BldB and its 10 paralogs. β-Galactosidase assays were performed to measure the strength of protein-protein interactions (in Miller units). The strong interaction between the leucine zipper domains of GCN4 (T18C-zip and T25-zip) was used as a positive control. Empty-vector negative control experiments were performed for every interaction displayed here, but for the sake of clarity, the results for only one representative negative control are displayed. Results are the average of three independent experiments, with two technical replicates per experiment. Error bars indicate the standard error of the mean.

The preceding results showed that *iosA* transcription was dramatically upregulated in the *bldB* mutant, that the transcription factor WhiJ9 was required for that activation, and that WhiJ9 mediates the activation of *iosA* expression by binding to direct repeats in the *iosA-whiJ9* intergenic region. This raised the possibility that hetero-oligomerization of BldB and BldB9 might somehow link BldB and the *iosA-whiJ9-bldB9* locus. To examine this possibility, we tested the BldB-BldB9 interaction in a bacterial two-hybrid (BACTH) system (34, 35). In these assays, the interaction of BldB with BldB9 gave β-galactosidase activities as high as the zip-zip positive control (the leucine zipper domains of GCN4; T18C-zip and T25-zip) and much higher than the interaction of BldB with itself (Fig. 8). This interaction could reflect heterodimerization or the formation of a higher-order complex such as a heterotetramer (dimer of dimers). We extended this analysis to examine the possible hetero-oligomerization of BldB with all 10 of its *S. venezuelae* paralogs. To ensure that none of the assayed proteins interacted with the T18 or T25 peptides, we performed empty-vector negative-control experiments. None of the negative-control results showed any interaction. For the sake of clarity, only one representative negative-control result is shown in Fig. 8. Like BldB9, many of the other paralogs interacted strongly with BldB, giving β-galactosidase activities comparable to those of the zip-zip positive control (Fig. 8). BldB2 and BldB10 showed a weaker interaction with BldB, but only BldB7 showed no interaction, giving β-galactosidase activities comparable to those of the negative controls (Fig. 8).

An amino acid sequence alignment of BldB and its paralogs in *S. venezuelae* is shown in Fig. S4A, and a maximum-likelihood phylogenetic tree constructed from the alignment is shown in Fig. S4B. The conserved regions in the alignment (KSSYSG, CVEVA, and VAVRDSKXP) are characteristic of the DUF397 domain shared by the BldB paralogs. However, BldB itself is a relatively poor match for this domain. It contains relatively few of the DUF397 conserved residues, and it has an extended N-terminal region that is absent in its *S. venezuelae* paralogs (Fig. S4A). Strikingly, the phylogenetic tree (Fig. S4B) shows that the BldB paralogs closest in sequence to canonical BldB, namely BldB7, BldB10, and BldB2, are the three paralogs that did not interact strongly with BldB in the BACTH assays (Fig. 8).

## DISCUSSION

Here, we have shown that loss of BldB function causes dramatic transcriptional upregulation of the *abaA* paralogs and a novel inhibitor of sporulation, *iosA*, and that cooverexpression of just two of these genes, *iosA* and *abaA6*, is sufficient to recapitulate the *bldB* mutant phenotype. We go on to show that the transcription factor WhiJ9 is required for the activation of *iosA* seen in the *bldB* mutant and that WhiJ9 mediates the activation of *iosA* expression by binding to direct repeats in the *iosA-whiJ9* intergenic region. We also show that BldB and BldB9 hetero-oligomerize, providing a potential link between BldB and the *iosA-whiJ9-bldB9* locus. Given that all five upregulated *abaA* paralogs lie next to a *whiJ* paralog, we speculate that their expression might also be regulated by the neighboring WhiJ transcription factor in a manner analogous to the regulation of *iosA* by WhiJ9. Further, the demonstrated ability of BldB to hetero-oligomerize with the BldB paralogs encoded adjacent to the *abaA* paralogs might similarly link BldB to regulatory events at those loci, creating a regulatory network. In that context, it is noticeable that *abaA7* and *abaA10*, the two *abaA* paralogs whose expression was least affected by deletion of *bldB* (Fig. 3), lie in gene clusters with *bldB7* and *bldB10* (Fig. 1), encoding BldB paralogs that did not interact strongly with BldB in the BACTH assays (Fig. 8). This would be consistent with the idea that hetero-oligomerization with a BldB paralog is required to allow BldB to affect expression of the cognate *abaA* gene. The third BldB paralog that did not interact strongly with BldB was BldB2 (Fig. 8), which is not clustered with an *abaA* paralog (Fig. 1). Although numerous questions remain to be answered, the work described here greatly expands our understanding of the global effects of the BldB developmental regulator.

The discovery of *iosA* as an inhibitor of sporulation that is dramatically upregulated in the *bldB* mutant is significant. However, the amino acid sequence of IosA is relatively uninformative. IosA is a predicted 15-kDa cytoplasmic protein with a pI of 10.3. We built a structural model of IosA using AlphaFold 2 (36) (see Fig. S5 in the supplemental material) and performed structural homology (Dali) searches, but the predicted structure of IosA is distinct from any previously solved structure in the protein database.

WhiJ9 likely directly activates the *iosA* promoter from its binding site in the *iosA-whiJ9* intergenic region, although we cannot formally exclude the possibility that this activation occurs by a more indirect route. The three direct repeats that constitute the WhiJ9 binding site lie 113 bp upstream of the *iosA* transcription start site, which is not a conventional location for a canonical activator. However, activation might occur through a looping mechanism, as often happens, for example, when bacterial enhancer binding proteins activate σ^54^ RNA polymerase from a distance (37, 38). Similar examples of remote activation are known for σ^70^-family σ factors. For example, Escherichia coli σ^70^ RNA polymerase is activated at the *papBA* promoter by catabolite gene activator protein (CAP) bound 215.5 bp upstream of the transcription start site (39). Further, there may be other, unidentified proteins involved with WhiJ9 in the activation of the *iosA* promoter. The WhiJ9 binding site lies downstream of the *whiJ9* promoter (Fig. 4A), suggesting that WhiJ9 should repress its own expression. It was possible to address this question because the constructed in-frame deletion of *whiJ9* did not remove the entire gene, being designed to delete only the first 159 amino acids of WhiJ9 and thus removing the DNA-binding domain but without disturbing the promoter of the downstream *bldB9* gene, which sits internal to the *whiJ9* coding sequence. As a consequence, there were RNA reads for *whiJ9* in the *whiJ9* mutant, and we could see that loss of WhiJ9 function caused a 2-fold increase in *whiJ9* transcription, consistent with autorepression.

In a search of 1,832 sequenced *Streptomyces* genomes, only 7 *Streptomyces* genomes appeared not to encode any BldB paralogs, suggesting that BldB paralogs are likely to play a conserved function throughout the genus. Based on the analysis of 100 actinobacterial genomes, it was previously claimed that BldB is confined to the genus *Streptomyces* (24). However, following the subsequent explosion of genome sequencing, it is clear from our expanded analysis that BldB is found in *Actinobacteria* outside the streptomycetes. In a search of 20,709 nonstreptomycete actinobacterial genomes, BldB paralogs were found encoded in 1,533 (7.4%). BldB was not found outside the *Actinobacteria*. Across the 3,358 *bldB*-containing actinobacterial genomes, the median number of *bldB* paralogs per genome was 19 (Fig. S6). At one end of the spectrum, 71 actinobacterial genomes were found to encode just a single BldB paralog. At the other end, the Actinomadura craniellae genome was found to encode 94 BldB paralogs. Most of the BldB paralogs identified in the searches consisted of just the BldB DUF397 domain and were therefore ≤100 amino acids long, like the 11 proteins found in *S. venezuelae*. However, some of the BldB paralogs identified were longer and carried additional domains. Notably, 129 of these longer BldB proteins represented fusions of BldB to the DNA-binding protein WhiJ, consistent with the single-domain BldB and WhiJ proteins having a collaborative function.

## MATERIALS AND METHODS

### Bacterial strains, plasmids, oligonucleotides, media, and conjugations.

The bacterial strains and plasmids used in this study are listed in Table S1, and oligonucleotides are listed in Table S2, both in the supplemental material. Escherichia coli strain DH5α was used for plasmid and cosmid manipulation and propagation. Disruption cosmids were generated using E. coli strain BW25113 (40) carrying a λ RED plasmid, pIJ790. The *dam dsm hsdS* E. coli strain ET12567 containing pUZ8002 (41) was used to conjugate cosmids and plasmids into *S. venezuelae* (42, 43). E. coli strains were grown on LB or LB agar at 37°C. *S. venezuelae* strains were grown in liquid or solid MYM supplemented with trace element solution (42) at 28°C. Where required for selection, the following antibiotics were added to the growth medium: 50 μg/mL apramycin, 100 μg/mL carbenicillin, 25 μg/mL chloramphenicol, 25 μg/mL hygromycin, and/or 50 μg/mL kanamycin.

### Construction and complementation of an *S. venezuelae bldB* null mutant.

Using “Redirect” PCR targeting (44, 45), *bldB* mutants in which the coding region was replaced with an apramycin resistance (*apr*) cassette were generated. An ordered cosmid library that covers >98% of the *S. venezuelae* genome (Maureen Bibb and Mark Buttner, unpublished) is fully documented at https://strepdb.streptomyces.org.uk/. Cosmid PL1_B10, carrying the *bldB* gene (*vnz26620*), was introduced into E. coli BW25113 containing pIJ790, and *bldB* was replaced with the *apr-oriT* cassette amplified from pIJ773 using the primer pair BldB_Redirect_F and BldB_Redirect_R. The resulting disrupted cosmid was confirmed by PCR analysis using flanking primers BldB_Red_Ext_F and BldB_Red_Ext_R and introduced into *S. venezuelae* by conjugation (43). Null mutant derivatives, generated by double crossover, were identified by their apramycin-resistant, kanamycin-sensitive, and morphological phenotypes, and their chromosomal structures were confirmed by PCR analysis using flanking primers BldB_Red_Ext_F and BldB_Red_Ext_R. Finally, the *bldB*::*apr* mutation was transduced back into WT *S. venezuelae* using the generalized transducing phage SV1 (46), and the chromosomal structures of the resulting mutants were again confirmed by PCR. A representative *bldB* null mutant was designated SV100. For complementation, *bldB* was amplified with primers BldB_Compl_F and BldB_Compl_R, generating a 617-bp fragment carrying the coding sequence and the *bldB* promoter, and cloned into HindIII/KpnI-cut pIJ10770. The plasmid was introduced into the *bldB* mutant by conjugation, integrating in *trans* at the ΦBT1 *attB* site, restoring WT levels of aerial mycelium formation and sporulation to the mutant.

### Construction of an *S. venezuelae* Δ*whiJ9* Δ*bldB* double mutant.

To construct a Δ*whiJ9* Δ*bldB* double mutant, a markerless *whiJ9* mutant was first constructed via CRISPR, using the pCRISPomyces-2 system (47). This in-frame deletion was designed to remove only the first 159 amino acids of WhiJ9, thus removing the DNA-binding domain, without disturbing the promoter of the downstream *bldB9* gene, which is internal to the *whiJ9* coding sequence. SV1-mediated generalized transduction was then used to introduce the *bldB*::*apr* mutation from the original Δ*bldB* mutant (SV100) into the unmarked Δ*whiJ9* background. Whole-genome resequencing was then used to confirm that no unintended mutations had occurred in the construction of the Δ*whiJ9* Δ*bldB* double mutant, which was designated SV125.

### Overexpression of genes in *S. venezuelae*.

Genes to be overexpressed in *S. venezuelae* were amplified using the respective OE_F and OE_R primer pairs described in Table S1 and cloned under the control of the strong constitutive ermE* promoter in the vector pIJ10257 (48), which integrates into the *S. venezuelae* chromosome at the ΦBT1 phage integration site. For overexpression of *abaA6* and *iosA* together, both amplified genes, separated by a short spacer containing a ribosome binding site, were inserted into NdeI/HindIII-digested pIJ10257 by Gibson Assembly (49), resulting in a construct that contained *abaA6* and *iosA* under the control of the same ermE* promoter. All overexpression plasmids were introduced into WT *S. venezuelae* by conjugation from E. coli (42, 43).

### Western blotting.

*S. venezuelae* cultures were grown in liquid MYM, and 2-mL samples were taken every 2 h from 10 h postinoculation onwards. Pellets were washed and resuspended in 0.4 mL of ice-cold sonication buffer (20 mM Tris HCL [pH 8.0], 5 mM EDTA, 1× EDTA-free protease inhibitor tablet [Roche, Basel, Switzerland]). Samples were sonicated at a 4.5-μm amplitude (5 cycles of 15 s on and 15 s off) on ice. Lysates were then centrifuged at 13,000 rpm for 15 min at 4°C to remove cell debris, and total protein concentrations were determined using Bradford assays. BldB levels were analyzed using the automated Wes Western blotting system (ProteinSimple, San Jose, CA) using a 1:500 dilution of an anti-BldB polyclonal antibody. Equal amounts of total protein (1.5 μg) were loaded for each sample, and results for each time point were analyzed in duplicate. The experiment was conducted according to the manufacturer's instructions, and results were analyzed using the CompassSW software.

### BACTH analysis.

BACTH screens were conducted using the adenylate cyclase system of Karimova et al. (34, 35). To construct the bait vectors, the *bldB* gene was amplified using primer pair BldB_BACTH_F and BldB_BACTH_R and cloned into plasmids pUT18C and pKNT25 digested with BamHI and KpnI to create pIJ10959 and pIJ10961. These plasmids express fusions of BldB with an N-terminal T18 and a C-terminal T25, respectively. To test for interactions with each of the 10 *bldB* paralogs from *S. venezuelae*, the *bldB2-bldB11* genes were amplified using the primer pairs described in Table S1 and cloned into the pKNT25 plasmid digested with BamHI and KpnI to create plasmids encoding the T25 domain of adenylate cyclase fused to the N terminus of each BldB paralog in turn (T25-BldB2 to T25-BldB11). E. coli BTH101 was cotransformed with pUT18C::*bldB* and the appropriate pKNT25 fusion plasmid. β-Galactosidase assays were conducted as described previously (42, 50).

### BldB ChIP-seq.

Cultures of WT *S. venezuelae* and the Δ*bldB* mutant strain were grown in 30-mL volumes of liquid MYM at 28°C, with shaking at 250 rpm. For this ChIP-seq experiment, duplicate WT *S. venezuelae* cultures from 3 different time points were prepared, with sampling at 10, 14, and 22 h postinoculation. Duplicate Δ*bldB* mutant cultures were sampled at 14 h and used as a negative control. To cross-link proteins bound to DNA, formaldehyde was added to the cultures at a final concentration of 1% (vol/vol), and incubation was continued for another 30 min. To stop the cross-linking reaction, glycine was then added to the cultures at a final concentration of 125 mM, and all cultures were incubated for 5 min at room temperature. The bacteria were harvested via centrifugation at 4°C at 7,000 rpm for 5 min and washed twice with ice-cold phosphate-buffered saline (PBS; pH 7.4). The resulting pellets were resuspended in 0.75 mL lysis buffer (10 mM Tris HCl [pH 8.0], 50 mM NaCl, with 10 mg/mL lysozyme and EDTA-free protease inhibitor [Roche, Basel, Switzerland] added) and incubated for 25 min at 37°C. Following lysis, an equal volume of immunoprecipitation (IP) buffer (100 mM Tris HCl [pH 8], 250 mM NaCl, 0.5% Triton X-100, 0.1% SDS, with protease inhibitor added) was added to the samples, which were then incubated on ice for 2 min. The samples were sonicated at an 8-μm amplitude (8 cycles of 20 s on and 1 min off) on ice, resulting in chromosomal DNA fragments ranging from 300 to 1,000 bp in size, centered on ~600 bp, as confirmed by agarose gel electrophoresis. The sonicated samples were centrifuged twice at 13,000 rpm at 4°C to remove debris, and the resulting supernatants were incubated with 10% (vol/vol) equilibrated 50% protein A-Sepharose (Sigma-Aldrich, Gillingham, UK) for 1 h on a rotating wheel, to remove nonspecifically bound proteins. Samples were then centrifuged at 13,000 rpm for 15 min to isolate the supernatants from the beads, and the supernatants were incubated with 10% (vol/vol) anti-BldB antibody overnight at 4°C on a rotating wheel. The following day, 10% (vol/vol) equilibrated 50% protein A-Sepharose was added to the samples and incubated for 4 h to recover the anti-BldB antibodies with bound protein and DNA. The samples were centrifuged for 5 min at 3,500 rpm at 4°C, the supernatant was discarded, and the pellets were washed twice with 0.5× IP buffer and then twice more with 1× IP buffer. To elute the DNA, the bead pellets were resuspended in 150 μL of IP elution buffer (50 mM Tris HCl [pH 7.6], 10 mM EDTA, 1% SDS) and incubated overnight at 65°C. The samples were then centrifuged for 5 min at 13,000 rpm to pellet the beads. The supernatants were retained, and the pellets were reextracted using 50 μL of TE buffer (10 mM Tris HCl [pH 7.4], 1 mM EDTA). The resulting supernatants were incubated with 3 μL of 10 mg/mL proteinase K for 2 h at 55°C to remove the bound proteins. Two phenol-chloroform extractions and one chloroform extraction were performed, and then the resulting DNA was further purified using a QiaQuick kit (Qiagen, Manchester, UK) and eluted in 50 μL of EB buffer (10 mM Tris HCl [pH 8.5]). Library construction and sequencing were performed by the Earlham Institute (Norwich, UK), using an Illumina HiSeq 2500.

ChIP-seq reads in the fastq files received from the sequencing contractors were aligned to the *S. venezuelae* genome (GenBank accession no. CP018074) using the most current version of bowtie2 software. This resulted in one SAM (.sam) file for each fastq file for all BldB ChIP-seq samples, for which single-ended sequencing was performed. For each SAM file, the “depth” command of “samtools” was used to arrive at the depth of sequencing at each nucleotide position of the *S. venezuelae* genome. From the sequencing depths at each nucleotide position determined in the previous step, a local enrichment was calculated in a moving window of 30 nucleotides (nt) moving in steps of 15 nt as (the mean depth at each nucleotide position in the 30-nt window) divided by (the mean depth at each nucleotide position in a 3,000-nt window centered around the 30-nt window). This resulted in an enrichment ratio value for every 15 nt along the genome. Enrichment for the control samples was subtracted from the enrichment for each sample.

### RNA isolation.

*S. venezuelae* cultures were grown in triplicate (for RNA-seq) or duplicate (for qRT-PCR) in liquid MYM. For the earliest time point (10 h postinoculation), 5 mL of culture was pelleted to gain enough biomass, whereas for the later time points, 2 mL of culture was pelleted and the medium supernatant was discarded. The pellets were washed in PBS and resuspended in 900 μL of lysis solution (400 μL of phenol at pH 4.3, 100 μL of chloroform-isoamyl alcohol [24:1], and 400 μL of RLT buffer [Qiagen, Manchester, UK]). The samples were transferred to lysing matrix B tubes (MP Biomedicals) and homogenized using a FastPrep FP120 cell disruptor (Thermo Savant). Two pulses of 30 s of intensity 6.0 were applied, with cooling on ice for 1 min between pulses. Supernatants were centrifuged for 15 min at full speed on a benchtop centrifuge at 4°C and then processed according to the instructions given in the RNeasy kit (Qiagen, Manchester, UK). The RNA samples were treated with on-column DNase I (Qiagen, Manchester, UK), followed by an additional DNase I treatment (Turbo DNA-free; Ambion). The RNA concentration was determined using a Qubit 2.0 fluorometer (Invitrogen).

### RNA-seq.

RNA library preparation and sequencing were performed by Genewiz (NJ, USA). rRNA depletion was conducted using the Illumina Ribo-Zero rRNA removal kit and paired-end sequencing conducted by Illumina HiSeq (2 × 150 bp configuration). The reads in the fastq files received from the sequencing contractor were aligned to the *S. venezuelae* genome (GenBank accession no. CP018074) using the most current version of bowtie2 software (51), which resulted in one SAM (.sam) file for each pair of fastq files (paired-end sequencing). The featureCounts() function of the Bioconductor package Rsubread was used to count the number of reads mapping to each gene on the chromosome (52). A quasi-likelihood F test implemented in the function glmQLFTest() of the Bioconductor package edgeR was used for differential expression analysis as described in the edgeR user's guide (53).

### qRT-PCR.

Equal amounts (1 μg) of total RNA from each sample was converted to cDNA using SuperScript II reverse transcriptase and random primers (Invitrogen). Two microliters of 10-fold-diluted cDNA was then used as a template in qRT-PCR. The experiment was conducted twice independently, and three technical replicates were performed for each of the two biological replicates. Primers iosA_qRT_F and iosA_qRT_R were used to amplify the *iosA* gene, and primers hrdB_qRT_F and hrdB_qRT_R were used to amplify the *hrdB* reference gene. The primers were designed using Primer3Plus with the following parameters: fragment size, 95 to 105 bp; primer length, 18 to 22 bp; melting temperature, 62 to 65°C. PCR using *S. venezuelae* genomic DNA was used to validate that only one product per primer pair was produced.

During each qRT-PCR experiment, a standard curve was constructed using serial dilutions of *S. venezuelae* chromosomal DNA to normalize for differing primer efficiencies. Melting curve analysis was used to confirm the production of a specific single product from each primer pair. qRT-PCR was performed using a CFX96 Touch deep-well real-time PCR detection system (Bio-Rad) instrument using Hard-Shell 96-well white PCR plates (Bio-Rad), sealed with thermostable film covers (ThermoFisher Scientific, Waltham, USA). The SensiFAST SYBR no-ROX kit (Bioline) was used to amplify PCR products according to the following protocol: 95°C for 3 min and then 45 cycles at 95°C for 5 s, 63°C for 10 s, and 72°C for 7 s. Melting curves were generated at 65°C to 95°C with 0.5°C increments. For the absolute quantification of target gene transcription, *Cq* (quantification cycle) values were calibrated using the genomic DNA standard curve for each primer pair, yielding the SQ (starting quantity) values for each sample. The *iosA* SQ values were divided by the mean *hrdB* SQ values for the corresponding samples, resulting in relative expression values. The mean relative expression values were normalized against the mean relative expression value of the WT, which was set to 1.

### Expression and purification of 6×His-WhiJ9.

Full-length *whiJ9* was amplified by PCR using primer pair WhiJ9_pET15B_F plus WhiJ9_pET15B_R and cloned into pET15b. The assembled plasmid was introduced into BL21(DE3) pLysS, and cultures were induced with 1 mM IPTG (isopropyl-β-d-thiogalactopyranoside) at 16°C overnight. All protein purification was conducted on an ÄKTA pure protein purification system (Cytiva, Marlborough, MA, USA). After lysis by sonication, the filtered supernatant was loaded onto a HisTrap high-performance or Excel 1-mL nickel column (Sigma-Aldrich, Gillingham, UK) equilibrated with buffer A (10 mM HEPES [pH 8], 250 mM NaCl, 50 mM imidazole). After washing with buffer A, bound protein was eluted from the column with buffer B (10 mM HEPES [pH 8], 250 mM NaCl, 500 mM imidazole). WhiJ9 fractions from the nickel column were pooled and concentrated using an Amicon Ultra-15 centrifugal filter unit with a 10-kDa cutoff (Merck, Gillingham, UK), flash-frozen in liquid nitrogen, and stored at −80°C.

### SPR.

SPR was performed using the ReDCaT method (32, 33). Single-stranded DNA oligonucleotides from target promoter regions were dissolved to a concentration of 100 μM. Complementary oligonucleotides were annealed to form ≤40-bp double-stranded DNA (dsDNA) molecules with a 20-bp single-stranded DNA (ssDNA) overhang (CCTACCCTACGTCCTCCTGC) complementary to the biotinylated ReDCaT linker. The annealed oligonucleotides were diluted to a concentration of 1 μM in running buffer (0.01 M HEPES [pH 7.4], 0.25 M NaCl, 3 mM EDTA, 0.05% [vol/vol] Tween 20).

A streptavidin sensor chip SA (Cytiva, Marlborough, MA, USA) was docked on a Biacore 8K system (Cytiva, Marlborough, MA, USA). A 20-bp biotinylated linker DNA was immobilized in the test flow cells of the chip. The DNA to be tested was then allowed to flow over this flow cell and the control cells at 10 μL/min to anneal to the single-stranded linker. 6×His-WhiJ9 was diluted to the desired concentration in running buffer and allowed to flow over both the reference control cell and the cell with immobilized DNA. The protein and test DNA were then washed away with 1 M NaCl–50 mM NaOH, regenerating the chip for loading of a new test DNA onto the immobilized linker. All experiments were performed at 25°C. Sensorgrams were generated using the Biacore 8K Insight Evaluation software (Cytiva, Marlborough, MA, USA). Results were normalized for different DNA molecules used by calculating the %*R*_max_ as previously described (33).

### SEM.

*S. venezuelae* colonies were mounted on the surface of an aluminum stub with optimal cutting temperature compound (Agar Scientific Ltd., Essex, UK), plunged into liquid nitrogen slush at approximately −210°C to cryopreserve the material, and transferred to the cryostage of an Alto 2500 cryotransfer system (Gatan, Oxford, UK) attached to an FEI Nova NanoSEM 450 (ThermoFisher Scientific, Eindhoven, The Netherlands). The surface frost was sublimated at −95°C for 3.5 min before the sample was sputter coated with platinum for 2 min at 10 mA at below −110°C. Finally, the sample was moved onto the cryostage in the main chamber of the microscope, held at approximately −125°C, and viewed at 3 kV.

### Data availability.

The RNA-seq transcriptional profiling data represented in Fig. 3 and 6 have been deposited in the MIAME-compliant ArrayExpress database (https://www.ebi.ac.uk/arrayexpress/) under accession numbers E-MTAB-12832 and E-MTAB-12836, respectively.
