# Supplementary material for: Global Effects of the Developmental Regulator BldB in Streptomyces venezuelae
Source: J Bacteriol. 2023 May 30;205(6):e00135-23. doi: 10.1128/jb.00135-23 (PMC10294661; doi:10.1128/jb.00135-23)
Supplement: Supplemental file 1 — Fig, S1 to S6 and Tables S1 and S2. Download jb.00135-23-s0001.pdf, PDF file, 0.6 MB [file jb.00135-23-s0001.pdf]

Supplemental material for

Global effects of the developmental regulator BldB in *Streptomyces venezuelae*

Marieta M. Avramova[a], Clare E.M. Stevenson[b], Govind Chandra[a], Neil A. Holmes[a], Matthew J. Bush[a], Kim C. Findlay[c], and Mark J. Buttner[a] *

[a]Department of Molecular Microbiology, [b]Department of Biochemistry and Metabolism, and [c]Department of Cell and Developmental Biology, John Innes Centre, Norwich Research Park, Norwich NR4 7UH, UK.

*Correspondence: mark.buttner@jic.ac.uk

This PDF file includes:

Figures S1 to S6

Tables S1 and S2

SM References

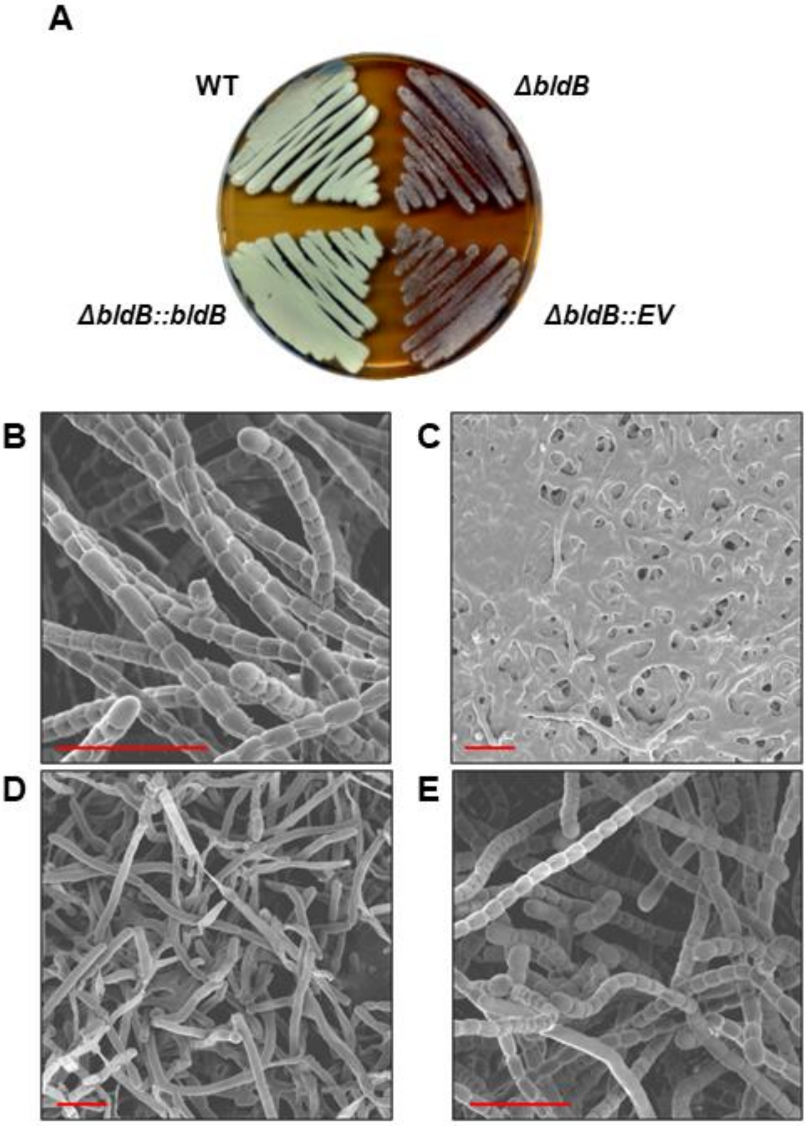

Fig. S1. Complementation of the *ΔbldB m*utant. The plate at the top (A) shows the phenotypes of WT *S. venezuelae*, the *bldB* mutant, the *bldB* mutant carrying the empty vector (EV; pIJ10770), and the complemented mutant, grown for 3 days at 28˚C on MYM agar. Below are scanning electron micrographs comparing the phenotypes of (B) wild-type *S. venezuelae*, (C) the colony centre of the *ΔbldB* mutant, (D) the colony periphery of the *ΔbldB* mutant, and (E) the complemented *ΔbldB* mutant. Scale bars in red indicate 5 µm.

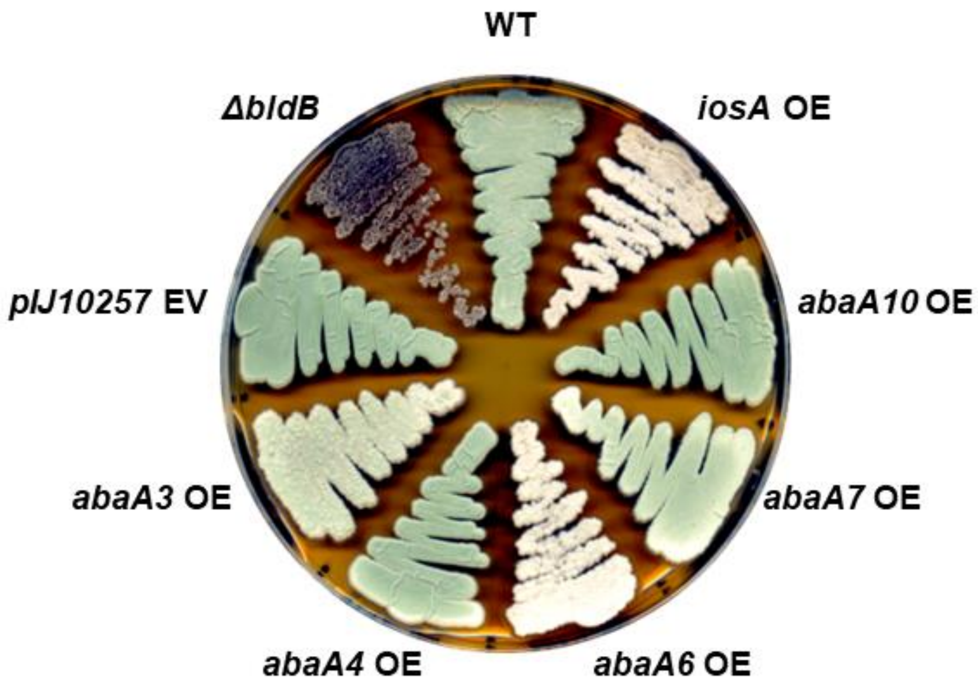

Fig. S2. Overexpression phenotypes for the *abaA* paralogs and *iosA*. Plate depicting the growth of the overexpression strains for the 5 *abaA* paralogs and for *iosA.* EV stands for the pIJ10257 empty vector control. The plate image was taken after 3 days of incubation at 28˚C on MYM agar.

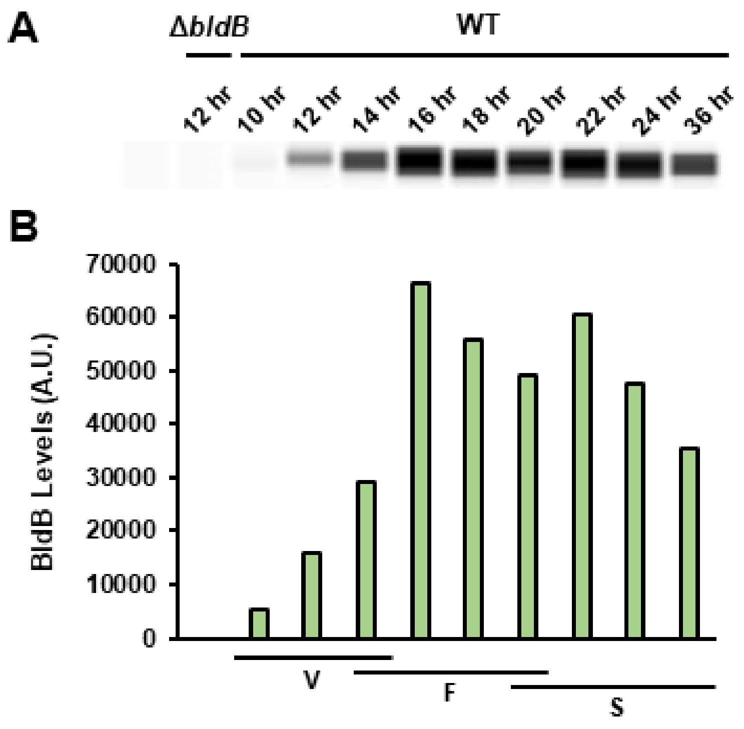

FIG S3. BldB Abundance Throughout Development. (A) Automated Western blot analysis showing BldB protein levels throughout development in MYM sporulation medium, generated using the quantitative 'Wes' capillary electrophoresis and blotting system (ProteinSimple – San Jose, CA; see Materials and Methods). Samples were taken every 2 hours from wild-type *S. venezuelae* during the vegetative (V), filamentous (F) and sporulation (S) growth stages. A sample from the Δ*bldB* mutant at 12 hours was used as a negative control. Equal amounts (1.5 µg) of total protein were loaded for each sample and BldB was detected with a polyclonal anti-BldB antibody. A single replicate is shown for each timepoint. (B) Quantification of BldB levels (area under each peak; arbitrary units [A.U.]). Bars represent the mean of two duplicates.

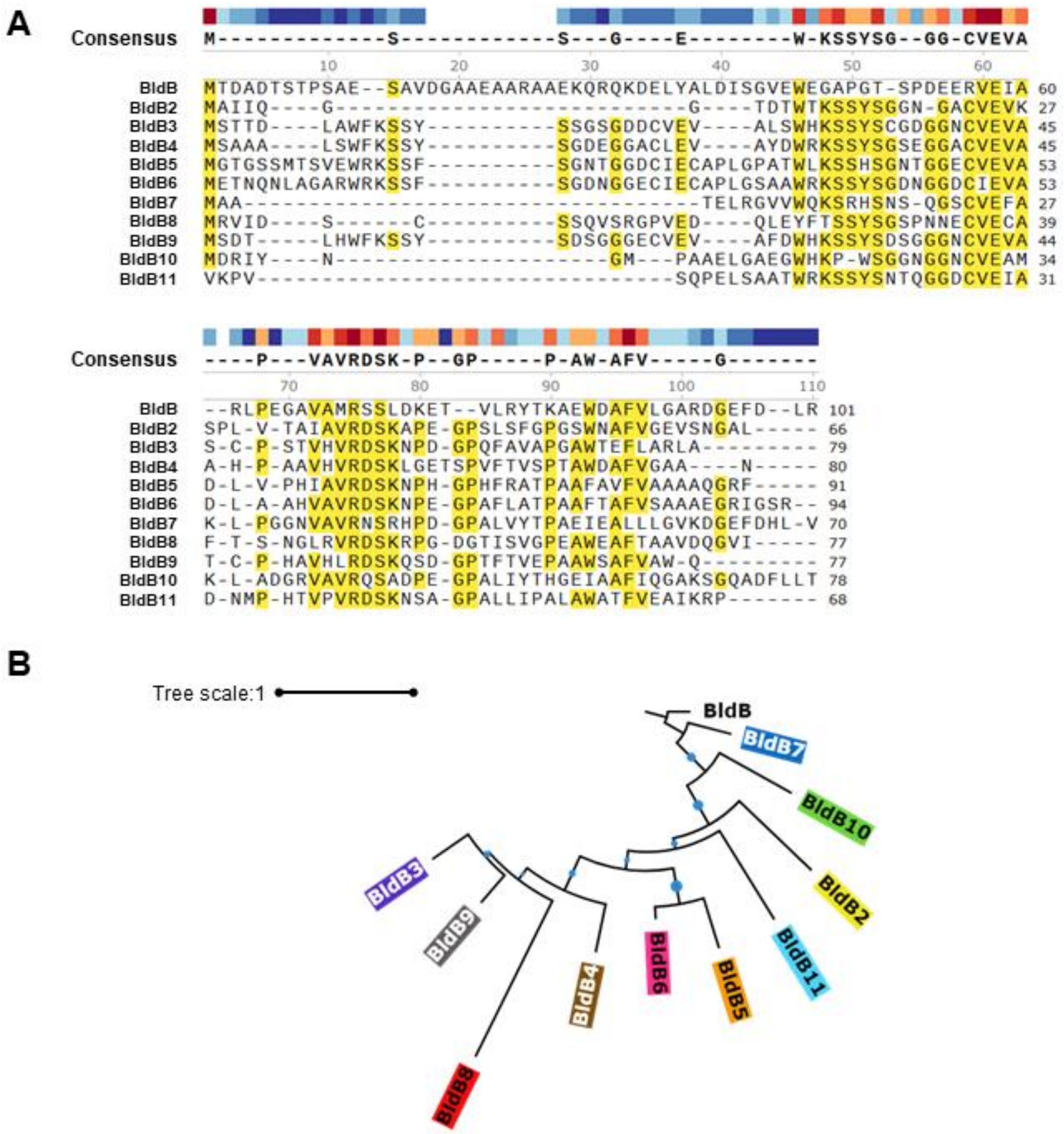

FIG S4. Relationship of Canonical BldB to its Paralogs in *S. venezuelae.* (A) Protein Sequence Alignment of BldB and its Paralogs. The amino acid sequences of the non-canonical BldB paralogs were aligned using T-Coffee. The sequence of canonical BldB was then aligned to this existing profile. The consensus sequence is displayed above, with high sequence identity regions marked in yellow. **(B) Phylogenetic Tree of BldB and its Paralogs.** A maximum-likelihood phylogeny of the *S. venezuelae* BldB paralogs. The sequence of canonical BldB was used to root the tree. Scale bar indicates the number of substitutions per site. The size of the circles on the branches indicates the number of bootstraps.

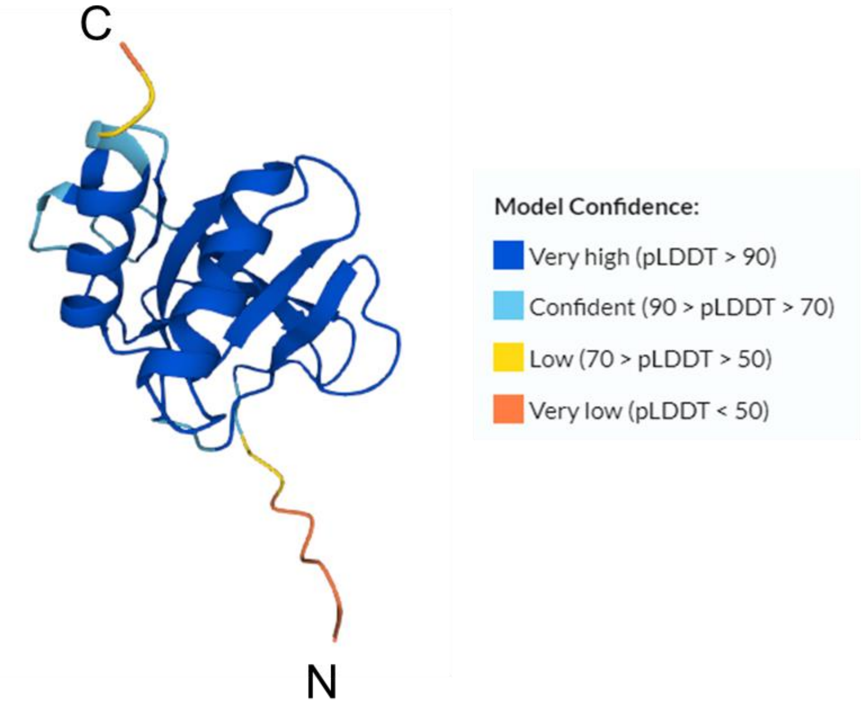

S. venezuelae IosA

MNEENREVNVDWIPVSGFQLRKAGVQFDAVRVDGDEGRRLASLMEALTGGDPGPVVIEANGRRGV
YFLVPPGSTARKAWPRGVTRFNGGPAHVSYVPVPALRGRTWPLSWRFPPTSPDRFVHPLLLRSAVTE
LFSGGS

Fig. S5. Structural Model of IosA. A structural model of IosA was built using AlphaFold 2 (Jumper *et al*., 2020). AlphaFold produces a per-residue confidence score (pLDDT) between 0 and 100 and the model color-coded accordingly. Some regions with low pLDDT may be unstructured in isolation.

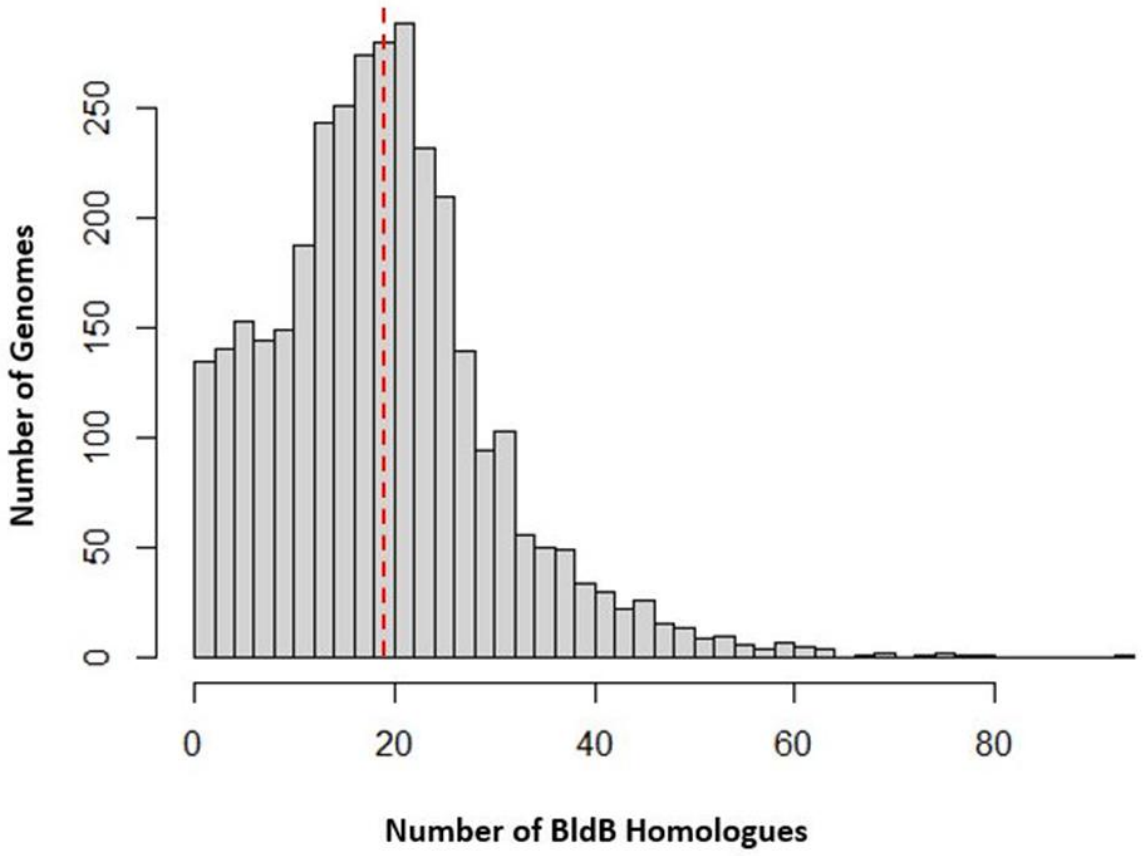

FIG S6. Number of BldB paralogs per genome. Histogram representing the number of BldB paralogs per genome in a survey of 3,358 *bldB*-containing Actinobacterial genomes. 71 were found to encode just a single *bldB* paralog. At the other end of the spectrum, *Actinomadura craniellae* was found to encode 94 *bldB* paralogs. Red dashed line indicates the median.

Table S1. Bacterial strains and plasmids used in this study.

Strain/phage	Genotype/comments	Source/reference
Streptomyces		
S. venezuelae NRRL B-65442	Wild type	Gomez-Escribano *et al*., 2021
SV100	Δ*bldB::apr* (Apr^R)	This study
SV125	Δ*whiJ9* Δ*bldB::apr* (Apr^R)	This study
SV1	Phage for generalized transduction in *S. venezuelae*	Stuttard, 1982; Smith *et al*., 2013
E. coli		
DH5α	F′ *supE44 ΔlacU169* (*Φ80lacZ ΔM15*) *ΔhsdR17 recA1 endA1 gyrA96 thi-1 relA1*	Hanahan, 1983
TOP10	*F⁻ mcrA Δ(mrr-hsdRMS-mcrBC) Φ80lacZΔM15 ΔlacX74 recA1 araD139 Δ(ara leu) 7697 galU galK rpsL (Str ^{R}) endA1 nupG*	ThermoFisher Scientific
BW25113/pIJ790	Δ(*araD-araB*)*567* Δ *lacZ4787*(::*rrnB-4*) *lacIp-4000*(*lacI^Q*), I-*rpoS369*(*Am*) *rph-1* Δ(*rhaD-rhaB*)*568 hsdR514*<br><br>pIJ790 is the λRED recombination plasmid	Datsenko and Wanner, 2000
ET12567/pUZ8002	F⁻ *dam13::Tn9 dcm6 hsdM hsdR recF143::Tn10 galK2 galT22 ara-14 lacY1 xyl-5 leuB6 thi-1 tonA31 rpsL hisG4 tsx-78 mtl-1 glnV44*<br><br>pUZ8002 is an RK2 derivative that is defective for self-mobilization, used as a driver plasmid (Kan^R)	Paget *et al*., 1999
BTH101	F⁻ *cya-99 araD139 galE15 galK16 rpsL1 (Str^r) hsdR2 mcrA1 mcrB1*	Karimova *et al*., 1998, 2000
BL21 DE3 pLysS Rosetta	F⁻ *ompT gal dcm lon hsd*S$_B$(r$_B^-$ m$_B^-$) λ(DE3 [*lac*I *lacUV5*-T7 gene 1 *ind*1 *sam*7 *nin*5]) pLysSRARE^6 (Clm^R)	Novagen (Merck, Nottingham, UK)

Plasmid	Genotype/comments	Source/reference
pIJ10770	pMS82 derivative with an intrinsic apramycin promoter removed and carrying an extended multiple cloning site (Hyg^R)	Schlimpert *et al*., 2017
pIJ10927	pIJ10770 carrying *bldB*	This study
pIJ10257	*Streptomyces* overexpression plasmid based on the strong *ermEp** promoter (Hyg^R). Integrates at the ΦBT1 *attB* site	Hong *et al*., 2005
pIJ10799	pCRISPomyces-2 (Cobb *et al,* 2015) carrying *whiJ9* flanking regions and a sgRNA internal to the *whiJ9* gene for the CRISPR-mediated markerless deletion of *whiJ9*	This study
pIJ10934	pIJ10257 carrying *bldB*	This study
pIJ10937	pIJ10257 carrying *abaA3*	This study
pIJ10938	pIJ10257 carrying *abaA4*	This study
pIJ10939	pIJ10257 carrying *abaA6*	This study
pIJ10940	pIJ10257 carrying *abaA7*	This study
pIJ10941	pIJ10257 carrying *abaA10*	This study
pIJ10942	pIJ10257 carrying *iosA*	This study
pIJ10944	pIJ10257 carrying *whiJ9*	This study

pIJ10950	pIJ10257 carrying *abaA6* and *iosA*	This study
pIJ10956	pET15B carrying *whiJ9* with an N-terminal 6xHis tag	This study
pIJ10959	pUT18C carrying *bldB*	This study
pIJ10961	pKNT25 carrying *bldB*	This study
pIJ10963	pKNT25 carrying *bldB2*	This study
pIJ10965	pKNT25 carrying *bldB3*	This study
pIJ10967	pKNT25 carrying *bldB4*	This study
pIJ10969	pKNT25 carrying *bldB5*	This study
pIJ10971	pKNT25 carrying *bldB6*	This study
pIJ10973	pKNT25 carrying *bldB7*	This study
pIJ10975	pKNT25 carrying *bldB8*	This study
pIJ10977	pKNT25 carrying *bldB9*	This study
pIJ10979	pKNT25 carrying *bldB10*	This study
pIJ10981	pKNT25 carrying *bldB11*	This study

**Cosmid**	**Genotype/comments**	**Source/reference**
PL1_B10	Used to generate a *ΔbldB* mutant in *S. venezuelae*	http://strepdb.streptomyces.org.uk/

Table S2. Oligonucleotides used in this study.

**Name**	**Sequence (5' - 3')**	**Digestion Sites**	**Purpose**
BldB_BACTH_F	ctgaggatcccATGACCGACGCAGACACCA	BamHI	Cloning *bldB* (*vnz26620*) into the BACTH vectors (PCR product is full gene without stop codon).
BldB_BACTH_R	ccggtacccgCCGCAGGTCGAACTCGCC	KpnI	
BldB2_BACTH_F	ctgaggatcccATGGCGATTATTCAGGGTGGCAC	BamHI	Cloning *bldB2* (*vnz15145*) into the BACTH vectors (PCR product is full gene without stop codon).
BldB2_BACTH_R	ccggtacccgGAGGGCCCCGTTGCTCAC	KpnI	
BldB3_BACTH_F	ctgaggatcccATGAGCACCACTGACCTGGCCT	BamHI	Cloning *bldB3* (vnz29075) into the BACTH vectors (PCR product is full gene without stop codon).
BldB3_BACTH_R	ccggtacccgCGCCAGCCGGGCGAGGAA	KpnI	
BldB4_BACTH_F	ctgaggatcccATGAGCGCAGCAGCACTTTCGT	BamHI	Cloning *bldB4* (*vnz25555*) into the BACTH vectors (PCR product is full gene without stop codon).
BldB4_BACTH_R	ccggtacccgGTTCGCCGCGCCGACGAA	KpnI	
BldB5_BACTH_F	ctgaggatcccATGGGGACCGGATCGAGCATGA	BamHI	Cloning *bldB5* (*vnz20565*) into the BACTH vectors (PCR product is full gene without stop codon).
BldB5_BACTH_R	ccggtacccgGAACCGCCCCTGCGCGG	KpnI	
BldB6_BACTH_F	ctgaggatcccATGGAGACCAACCAGAACCTGGC	BamHI	Cloning *bldB6* (*vnz09895*) into the BACTH vectors (PCR product is full gene without stop codon).
BldB6_BACTH_R	ccggtacccgGCGACTGCCGATGCGCCC	KpnI	
BldB7_BACTH_F	AACTGCAGCGTGCATCATGCGCATCGTGCG	PstI	Cloning *bldB7* (*vnz16140*) into the BACTH vectors (PCR product is full gene without stop codon).
BldB7_BACTH_R	ccggtacccgCACCAGGTGGTCGAACTCCC	KpnI	

Name	Sequence (5' - 3')	Digestion Sites	Purpose
BldB8_BACTH_F	ctgaggatcccGTGGAAGACCAGCTCGAATACT	BamHI	Cloning *bldB8* (*vnz28285*) into the BACTH vectors (PCR product is full gene without stop codon).
BldB8_BACTH_R	ccggtacccgGATCACGCCTTGATCCACCG	KpnI	
BldB9_BACTH_F	ctgaggatcccATGAGCGACACCCTGCACTG	BamHISDD	Cloning *bldB9* (*vnz16680*) into the BACTH vectors (PCR product is full gene without stop codon).
BldB9_BACTH_R	ccggtacccgCTGCCACGCGACGAAAGC	KpnI	
BldB10_BACTH_F	ctgaggatcccATGGATCGCATATACAACGGCA	BamHI	Cloning *bldB10* (*vnz31505*) into the BACTH vectors (PCR product is full gene without stop codon).
BldB10_BACTH_R	ccggtacccgGGTGAGCAGAAAGTCAGCC	KpnI	
BldB11_BACTH_F	ctgaggatcccGTGAAGCCCGTGTCCCAACC	BamHI	Cloning *bldB11* (*vnz28375*) into the BACTH vectors (PCR product is full gene without stop codon).
BldB11_BACTH_R	ccggtacccgCGGGCGCTTGATCGCCTC	KpnI	
WhiJ9_pET15B_F	GCGGCCTGGTGCCGCGCGGCAGCCACATGGCGCGTGCGGAGAACAAGG		Overexpression of *whiJ9* in *E. coli* for protein purification. PCR product was cloned into pET15B via Gibson assembly.
WhiJ9_pET15B_R	TCGGGCTTTGTTAGCAGCCGGATCCTCATCTTTGTTCTCCCGCCAACTCC		
BldB_Redirect_F	GAGGGACCGCCGTACCACCGCGAAGGGAACGCGCCGATGATTCCGGGGATCCGTCGACC		Redirect deletion of *bldB* (*vnz26620*).
BldB_Redirect_R	CGTACGACGGGCCACCCCCTCACGCGTGTCCGCGGCTCATGTAGGCTGGAGCTGCTTC		
BldB_Red_Ext_F	ACATCCTCGGCAACGTCCGG		External primers for confirmation of the Redirect deletion of *bldB* (*vnz26620*).
BldB_Red_Ext_R	CCCAGCTCCTCGACGAGTC		
WhiJ9_CRISPR_Frag1_F	tgccgccgggcgttttttatCGCATCGAACCGGGCCACGC		Amplification and cloning of repair flanking regions for *whiJ9* in pCRISPomyces-2 via Gibson assembly.
WhiJ9_CRISPR_Frag1_R	GACTCGTCGAGGATCAGCTCCATTCACGTTCCCCATTCGGAC		
WhiJ9_CRISPR_Frag2_F	CCGAATGGGGAACGTGAATGGAGCTGATCCTCGACGAGTCGG		
WhiJ9_CRISPR_Frag2_R	gcggcctttttacggttcctggcctCGCAAGCTGGTCACCTCACTG		
WhiJ9_sgRNA_F1	ACGCCGCCCAGGACGCGTTCGAGC	BbsI	Cloning of a protospacer for *whiJ9* in pCRISPomyces-2 via Golden Gate cloning.
WhiJ9_sgRNA_R1	AAACGCTCGAACGCGTCCTGGGCG	BbsI	
WhiJ9_sgRNA_F2	ACGCGCCACCGCCTTCTGTTCGAT	BbsI	Cloning of a protospacer for *whiJ9* in pCRISPomyces-2 via Golden Gate cloning.
WhiJ9_sgRNA_R2	AAACATCGAACAGAAGGCGGTGGC	BbsI	
BldB_Compl_F	GCAGAAGCTTGACGTCGGCATCCGCTTCAT	HindIII	Complementation of the Δ*bldB* mutant with *bldB in trans*. PCR product was cloned into pIJ10770.
BldB_Compl_R	GGCGGTACCGCTCACCGCAGGTCGAACT	KpnI	
BldB_OE_F	GGGAATTCCATATGACCGACGCAGACACCAG	NdeI	Overexpression of *bldB (vnz26620)* in *S. venezuelae*. PCR product was cloned into pIJ10257.
BldB_OE_R	TCCAAGCTTTCACCGCAGGTCGAACTCG	HindIII	
AbaA3_OE_F	GGGAATTCCATATGACCTCCCCCGTGACCC	NdeI	Overexpression of *abaA3 (vnz29085)* in *S. venezuelae*. PCR product was cloned into pIJ10257.
AbaA3_OE_R	GTCCAAGCTTTCAGGGAACCACGAGGTCGAG	HindIII	

Name	Sequence (5' - 3')	Digestion Sites	Purpose
AbaA4_OE_F	GGGAATTCCATATGAGCGACGAACT CCCCCTG	NdeI	Overexpression of *abaA4 (vnz25565)* in *S. venezuelae*. PCR product was cloned into pIJ10257.
AbaA4_OE_R	GTCCAAGCTTCTAGGGGCTGTCCGG GAAG	HindIII	
AbaA6_OE_F	GGGAATTCCATATGAATCAGGCAACA GACCTCT	NdeI	Overexpression of *abaA6 (vnz09905)* in *S. venezuelae*. PCR product was cloned into pIJ10257.
AbaA6_OE_R	GTCCAAGCTTCTACGCGTCGCACTC GAA	HindIII	
AbaA7_OE_F	GGGAATTCCATATGGGGACGAATGG ATCGACC	NdeI	Overexpression of *abaA7 (vnz16135)* in *S. venezuelae*. PCR product was cloned into pIJ10257.
AbaA7_OE_R	GTCCAAGCTTCTACTCCGGGCCGAT CCG	HindIII	
AbaA10_OE_F	CCGCTCGAGATGGCCCAGCGCCCG CAG	XhoI	Overexpression of *abaA10 (vnz31495)* in *S. venezuelae*. PCR product was cloned into pIJ10257.
AbaA10_OE_R	GTCCAAGCTTTCAGCACGGTTCCCC CATGC	HindIII	
IosA_OE_F	GGGAATTCCATATGGTGAATGAAGA GAATCGGGAAGT	NdeI	Overexpression of *iosA (vnz16670)* in *S. venezuelae*. PCR product was cloned into pIJ10257.
IosA_OE_R	GTCCAAGCTTTCAGGACCCGCCGCT GAA	HindIII	
WhiJ9_OE_F	GGGAATTCCATATGGCGCGTGCGGA GAACA	NdeI	Overexpression of *whiJ9 (vnz16675)* in *S. venezuelae*. PCR product was cloned into pIJ10257.
WhiJ9_OE_R	GTCCAAGCTTTCATCTTTGTTCTCCC GCCAAC	HindIII	
AbaA6_IosA_2xOE _Frag1_F	gtctagaacaggaggccccatATGAATCAGG CAACAGACCTCTAT		Tandem overexpression of *abaA6 (vnz09905) and iosA (vnz16670)* in *S. venezuelae*. PCR product was cloned into pIJ10257 via Gibson assembly. An RBS was placed between the two genes.
AbaA6_IosA_2xOE _Frag1_R	ACggcttacctccgatgttgagCTACGCGTCG CACTCGAACCACA		
AbaA6_IosA_2xOE _Frag2_F	TAGctcaacatcggaggtaagccGTGAATGA AGAGAATCGGGAAGT		
AbaA6_IosA_2xOE _Frag2_R	tgagaaccctaggggatccaTCAGGACCCGC CGCTGAACAGC		
IosA_qRT_F	GACCTGGCCGCTGTCCTG		For amplification of a fragment of the *iosA* (*vnz16670*) gene in qRT-PCR.
IosA_qRT_R	GGACCCGCCGCTGAACAG		
HrdB_qRT_F	TGTTCTGCGCAGCCTCAATCAG		For amplification of a fragment of the *hrdB* gene in qRT-PCR.
HrdB_qRT_R	CTCTTCGCTGCGACGCTCTT		
O1_ReDCaT_F	TCACGTTCCCCATTCGGACTCAACC GATCTCAACCGGTCT		Testing if WhiJ9 binds overlapping dsDNA oligos from the *iosA-whiJ9* intergenic region via ReDCaT.
O1_ReDCaT_R	AGACCGGTTGAGATCGGTTGAGTCC GAATGGGGAACGTGAcctaccctacgtcctc ctgc		
O2_ReDCaT_F	GATCTCAACCGGTCTCAACTGCTCC CCGCCGAATTCGACT		
O2_ReDCaT_R	AGTCGAATTCGGCGGGGAGCAGTTG AGACCGGTTGAGATCcctaccctacgtcctc ctgc		
O3_ReDCaT_F	CCGCCGAATTCGACTGCACACGAGG GGCGTTGGTGCAGGT		
O3_ReDCaT_R	ACCTGCACCAACGCCCCTCGTGTGC AGTCGAATTCGGCGGcctaccctacgtcctc ctgc		

Name	Sequence (5' - 3')	Digestion Sites	Purpose
O4_ReDCaT_F	GGCGTTGGTGCAGGTCGAGGGGTTACCGACGCGCCCGCCA		Testing if WhiJ9 binds overlapping dsDNA oligos from the *iosA-whiJ9* intergenic region via ReDCaT.
O4_ReDCaT_R	TGGCGGGCGCGTCGGTAACCCCTCGACCTGCACCAACGCCcctaccctacgtcctcctgc		
O5_ReDCaT_F	CCGACGCGCCCGCCACAGTCGTGGAAAACGCTAGCGCCGG		
O5_ReDCaT_R	CCGGCGCTAGCGTTTTCCACGACTGTGGCGGGCGCGTCGGcctaccctacgtcctcctgc		
O6_ReDCaT_F	GTCGTGGAAAACGCTAGCGCCGGCGCGGGAACATGTCCTC		
O6_ReDCaT_R	GAGGACATGTTCCCGCGCCGGCGCTAGCGTTTTCCACGACcctaccctacgtcctcctgc		
LH1_ReDCaT_F	GTTCCCCATTCGGACTCAACCGATCTCAACCGGTCTCAAC		Finding a minimal binding site for WhiJ9 in the *iosA-whiJ9* intergenic region via ReDCaT footprinting.
LH1_ReDCaT_R	GTTGAGACCGGTTGAGATCGGTTGAGTCCGAATGGGGAACcctaccctacgtcctcctgc		
LH2_ReDCaT_F	TCCCCATTCGGACTCAACCGATCTCAACCGGTCTCAAC		
LH2_ReDCaT_R	GTTGAGACCGGTTGAGATCGGTTGAGTCCGAATGGGGAcctaccctacgtcctcctgc		
LH3_ReDCaT_F	CCCATTCGGACTCAACCGATCTCAACCGGTCTCAAC		
LH3_ReDCaT_R	GTTGAGACCGGTTGAGATCGGTTGAGTCCGAATGGGcctaccctacgtcctcctgc		
LH4_ReDCaT_F	CATTCGGACTCAACCGATCTCAACCGGTCTCAAC		
LH4_ReDCaT_R	GTTGAGACCGGTTGAGATCGGTTGAGTCCGAATGcctaccctacgtcctcctgc		
LH5_ReDCaT_F	TTCGGACTCAACCGATCTCAACCGGTCTCAAC		
LH5_ReDCaT_R	GTTGAGACCGGTTGAGATCGGTTGAGTCCGAAcctaccctacgtcctcctgc		
LH6_ReDCaT_F	CGGACTCAACCGATCTCAACCGGTCTCAAC		
LH6_ReDCaT_R	GTTGAGACCGGTTGAGATCGGTTGAGTCCGcctaccctacgtcctcctgc		
LH7_ReDCaT_F	GACTCAACCGATCTCAACCGGTCTCAAC		
LH7_ReDCaT_R	GTTGAGACCGGTTGAGATCGGTTGAGTCcctaccctacgtcctcctgc		
LH8_ReDCaT_F	CTCAACCGATCTCAACCGGTCTCAAC		
LH8_ReDCaT_R	GTTGAGACCGGTTGAGATCGGTTGAGcctaccctacgtcctcctgc		
LH9_ReDCaT_F	CAACCGATCTCAACCGGTCTCAAC		
LH9_ReDCaT_R	GTTGAGACCGGTTGAGATCGGTTGcctaccctacgtcctcctgc		
LH10_ReDCaT_F	ACCGATCTCAACCGGTCTCAAC		
LH10_ReDCaT_R	GTTGAGACCGGTTGAGATCGGTcctaccctacgtcctcctgc		

Name	Sequence (5' - 3')	Digestion Sites	Purpose
LH11_ReDCaT_F	CGATCTCAACCGGTCTCAAC		Finding a minimal binding site for WhiJ9 in the *iosA-whiJ9* intergenic region via ReDCaT footprinting.
LH11_ReDCaT_R	GTTGAGACCGGTTGAGATCGcctaccctacgtcctcctgc		
LH12_ReDCaT_F	ATCTCAACCGGTCTCAAC		
LH12_ReDCaT_R	GTTGAGACCGGTTGAGATcctaccctacgtcctcctgc		
LH13_ReDCaT_F	CTCAACCGGTCTCAAC		
LH13_ReDCaT_R	GTTGAGACCGGTTGAGcctaccctacgtcctcctgc		
RH1_ReDCaT_F	CGGACTCAACCGATCTCAACCGGTCTCAACTGCTCCCCGC		
RH1_ReDCaT_R	cctaccctacgtcctcctgcGCGGGGAGCAGTTGAGACCGGTTGAGATCGGTTGAGTCCG		
RH2_ReDCaT_F	CGGACTCAACCGATCTCAACCGGTCTCAACTGCTCCCC		
RH2_ReDCaT_R	cctaccctacgtcctcctgcGGGGAGCAGTTGAGACCGGTTGAGATCGGTTGAGTCCG		
RH3_ReDCaT_F	CGGACTCAACCGATCTCAACCGGTCTCAACTGCTCC		
RH3_ReDCaT_R	cctaccctacgtcctcctgcGGAGCAGTTGAGACCGGTTGAGATCGGTTGAGTCCG		
RH4_ReDCaT_F	CGGACTCAACCGATCTCAACCGGTCTCAACTGCT		
RH4_ReDCaT_R	cctaccctacgtcctcctgcAGCAGTTGAGACCGGTTGAGATCGGTTGAGTCCG		
RH5_ReDCaT_F	CGGACTCAACCGATCTCAACCGGTCTCAACTG		
RH5_ReDCaT_R	cctaccctacgtcctcctgcCAGTTGAGACCGGTTGAGATCGGTTGAGTCCG		
RH6_ReDCaT_F	CGGACTCAACCGATCTCAACCGGTCTCAAC		
RH6_ReDCaT_R	cctaccctacgtcctcctgcGTTGAGACCGGTTGAGATCGGTTGAGTCCG		
RH7_ReDCaT_F	CGGACTCAACCGATCTCAACCGGTCTCA		
RH7_ReDCaT_R	cctaccctacgtcctcctgcTGAGACCGGTTGAGATCGGTTGAGTCCG		
RH8_ReDCaT_F	CGGACTCAACCGATCTCAACCGGTCT		
RH8_ReDCaT_R	cctaccctacgtcctcctgcAGACCGGTTGAGATCGGTTGAGTCCG		
RH9_ReDCaT_F	CGGACTCAACCGATCTCAACCGGT		
RH9_ReDCaT_R	cctaccctacgtcctcctgcACCGGTTGAGATCGGTTGAGTCCG		
RH10_ReDCaT_F	CGGACTCAACCGATCTCAACCG		
RH10_ReDCaT_R	cctaccctacgtcctcctgcCGGTTGAGATCGGTTGAGTCCG		
RH11_ReDCaT_F	CGGACTCAACCGATCTCAAC		
RH11_ReDCaT_R	cctaccctacgtcctcctgcGTTGAGATCGGTTGAGTCCG		
RH12_ReDCaT_F	CGGACTCAACCGATCTCA		
RH12_ReDCaT_R	cctaccctacgtcctcctgcTGAGATCGGTTGAGTCCG		

Name	Sequence (5' - 3')	Digestion Sites	Purpose
RH13_ReDCaT_F	CGGACTCAACCGATCT		
RH13_ReDCaT_R	cctaccctacgtcctcctgcAGATCGGTTGAG TCCG		
IosA_WhiJ9_ DR123_F	TACGGACTCAACCGATCTCAACCGG TCTCAACAT		Characterisation of WhiJ9 binding to direct repeats in the *iosA-whiJ9* intergenic region via ReDCaT.
IosA_WhiJ9_ DR123_R	ATGTTGAGACCGGTTGAGATCGGTT GAGTCCGTAcctaccctacgtcctcctgc		
IosA_WhiJ9_ DR12_F	TACGGACTCAACCGATCTCAACCATG CAGCGGAT		
IosA_WhiJ9_ DR12_R	ATCCGCTGCATGGTTGAGATCGGTT GAGTCCGTAcctaccctacgtcctcctgc		
IosA_WhiJ9_ DR23_F	TACATGCAGCGGCGATCTCAACCGG TCTCAACAT		
IosA_WhiJ9_ DR23_R	ATGTTGAGACCGGTTGAGATCGCCG CTGCATGTAcctaccctacgtcctcctgc		
IosA_WhiJ9_ DR13_F	TACGGACTCAACCATGCAGCGGCGG TCTCAACAT		
IosA_WhiJ9_ DR13_R	ATGTTGAGACCGCCGCTGCATGGTT GAGTCCGTAcctaccctacgtcctcctgc		
IosA_WhiJ9_ DR1only_F	TACGGACTCAACCATGCAGCGGATG AGGGAGCAT		
IosA_WhiJ9_ DR1only_R	ATGCTCCCTCATCCGCTGCATGGTTG AGTCCGTAcctaccctacgtcctcctgc		
IosA_WhiJ9_ DR2only_F	TACATGCAGCGGCGATCTCAACATG AGGGAGCAT		
IosA_WhiJ9_ DR2only_R	ATGCTCCCTCATGTTGAGATCGCCG CTGCATGTAcctaccctacgtcctcctgc		
IosA_WhiJ9_ DR3only_F	TACATGCAGCGGATGAGGGAGCCGG TCTCAACAT		
IosA_WhiJ9_ DR3only_R	ATGTTGAGACCGGCTCCCTCATCCG CTGCATGTAcctaccctacgtcctcctgc		

SM References

Datsenko KA, Wanner BL. 2000. One-step inactivation of chromosomal genes in *Escherichia coli* K-12 using PCR products. Proc Natl Acad Sci USA 97:6640–6645.

Gomez-Escribano JP, Holmes NA, Schlimpert S, Bibb MJ, Chandra G, Wilkinson B, Buttner MJ, Bibb MJ. 2021. *Streptomyces venezuelae* NRRL B-65442: genome sequence of a model strain used to study morphological differentiation in filamentous actinobacteria', J Ind Microbiol Biotechnol 48:kuab035.

Hanahan D. 1983. Studies on transformation of *Escherichia coli* with plasmids. J Mol Biol 166:557–580.

Hong H-J, Hutchings MI, Hill LM, Buttner MJ. 2005. The role of the novel Fem protein VanK in vancomycin resistance in *Streptomyces coelicolor*. J Biol Chem 280:13055-13061.

Jumper J, *et al.* 2021. Highly accurate protein structure prediction with AlphaFold. Nature 596: 583–589.

Karimova G, Pidoux J, Ullmann A, Ladant D. 1998. A bacterial two-hybrid system based on a reconstituted signal transduction pathway', Proc Natl Acad Sci USA 95:5752–5756.

Karimova G, Ullmann A, Ladant D. 2000. *Bordetella pertussis* adenylate cyclase toxin as a tool to analyze molecular interactions in a bacterial two-hybrid system. Int Journal Med Microbiol 290:441–445.

Paget MS, Chamberlin L, Atrih A, Foster SJ, Buttner MJ. 1999. Evidence that the extracytoplasmic function sigma factor σ^E is required for normal cell wall structure in *Streptomyces coelicolor* A3(2). J Bacteriol 181:204–11.

Schlimpert S, Wasserstrom S, Chandra G, Bibb MJ, Findlay KC, Flärdh K, Buttner MJ. 2017. Two dynamin-like proteins stabilize FtsZ rings during *Streptomyces* sporulation. Proc Natl Acad Sci USA 114:E6176-E6183.

Smith MCM, Hendrix RW, Dedrick R, Mitchell K, Ko C-C, Russell D, Bell E, Gregory M, Bibb MJ, Pethick F, Jacobs-Sera D, Herron P, Buttner MJ, Hatfull GF. 2013. Evolutionary relationships within actinophages and a putative adaptation for growth in *Streptomyces* spp. J Bacteriol 195:4924-4935.

Stuttard C. 1979. Transduction of auxotrophic markers in a chloramphenicol-producing strain of *Streptomyces*. J Gen Microbiol 110:479–482.
